# Healthcare access, quality and financial risk protection among displaced Venezuelan women living in Brazil: a cross-sectional study

**DOI:** 10.1016/j.lana.2024.100830

**Published:** 2024-06-29

**Authors:** Rodrigo Moreno-Serra, Ivan Ochoa-Moreno, Misael Anaya-Montes, Luis Cardoso Fernandes, Thaiza Gomes, Maria Do Carmo Leal, Cristóbal Cuadrado

**Affiliations:** a Centre for Health Economics, University of York, UK; b Universidad Nacional del Callao, Peru; c Janssen Pharmaceutica N.V., Beerse, Belgium; d Fundação Oswaldo Cruz, Rio de Janeiro, Brazil; e Escuela de Salud Pública, Universidad de Chile, Santiago, Chile

**Keywords:** Forced displaced populations, Universal healthcare coverage, Financial risk protection, Healthcare access, Quality of care

## Abstract

**Background:**

Millions of Venezuelans have been displaced because of deteriorating socio-economic conditions in their country. We examine key domains of universal health coverage among migrant Venezuelan women in Brazil: healthcare access, care quality and financial risk protection.

**Methods:**

We collected primary data on 2012 Venezuelan women aged 15–49 who migrated to Brazil between 2018 and 2021, in the cities of Boa Vista and Manaus, along with secondary data for Brazilian women. We used linear regression with entropy balance matching to estimate associations between migrant status and healthcare spending, utilisation and quality indicators.

**Findings:**

Our sample had a mean age of 29.5 years (S.D. 8.9), 64% (1286/2011) of mixed ethnicity, 29% (577/2011) white, 4% (71/2011) black, 3% (62/2011) indigenous and 1% (15/2011) other ethnicities. Compared to Brazilian women, migrant women had 9.5 percentage points (pp) (95% CI: 6 pp–13 pp; p < 0.0001) higher catastrophic health expenditure incidence. Migrants were 27 pp (95% CI: 11 pp–43 pp; p = 0.0008) more likely to receive healthcare when sought, but 37 pp (95% CI: −45 pp to −29 pp; p < 0.0001) less likely to have had a pap smear in the last three years. Migrants were as likely as non-migrants to have received pap smear results within three months (95% CI: −9 pp to 22 pp; p = 0.39) and clinically appropriate antenatal consultations (95% CI: −10 pp to 40 pp; p = 0.23).

**Interpretation:**

Migrant women in Brazil have relatively good healthcare access and quality outcomes. Yet a potential backlog of unmet sexual and reproductive healthcare needs and inadequate financial risk protection require policy attention.

**Funding:**

UK 10.13039/501100000269Economic and Social Research Council (ES/T00441X/1).

**Research in context**

**Evidence before this study**

We searched Pub Med and Google Scholar for health challenges imposed by forced displacement in some South–South migration contexts from a gendered viewpoint. We used the following searching strategy: (refugee OR refugees OR asylum OR displace∗ OR (forced AND migra∗)) AND health. Our search was limited to articles published up to 2021. We identified 1454 articles, from which a total of 43 studies focused on the causal impact of forced displacement on maternal and child health outcomes. These studies have largely adopted a descriptive methodological perspective, focusing on access issues among migrants recently arrived in the host community. Even w here studies have focused on the inter-relationships between forced displacement and broader health indicators, important methodological challenges (e.g. lack of adequate comparison groups for displaced individuals) make it difficult to draw firm conclusions from findings.

**Added value of this study**

Our study provides a novel assessment of universal health coverage indicators—related to healthcare access, quality and financial risk protection—for two populations of displaced Venezuelan women in Brazil, by contrasting such information with data for comparable Brazilian women. Our estimations reveal relatively good healthcare access and care quality conditions among migrant women when compared to non-migrants, but there is evidence suggesting a potential backlog of unmet sexual and reproductive healthcare needs and high incidence of catastrophic healthcare payments among Venezuelan migrants.

**Implications of all the available evidence**

Venezuelan migrant women have access to quality healthcare in the Brazilian public health system, *Sistema Único de Saúde* (SUS). However, the higher incidence of catastrophic healthcare payments among these migrants, compared to Brazilian women, raises questions about why SUS—a publicly funded universal health system—seems to be facing challenges to ensuring adequate standards of financial risk protection for Venezuelan women. Policy action is needed to avoid the deterioration of gender gaps and poverty among migrant women in Brazil that may arise from inadequate protection against unaffordable payments for healthcare.

## Introduction

Venezuela has experienced a long economic recession and high inflation rates over the last decade, prompting a large-scale humanitarian crisis.^1^ The situation poses a particular threat for women and children, reflected in increasing rates of violence against women and child mortality surpassing 26 per thousand live births.^1^

Consequently, Venezuela has seen a massive outflow of citizens to neighbouring countries since 2014. Estimates from September 2019 suggest that 4.3 million Venezuelans, nearly 13.6 percent of its 2016 population, left their country for Colombia, Ecuador, Brazil and others, in what has been globally recognised as massive forced displacement.^2^ Brazil has seen a significant influx of Venezuelans particularly to its northern state of Roraima. As of 2018, displaced Venezuelans in Roraima accounted for nearly 40% of all asylum requests by Venezuelans in Brazil.^3^^,^^4^

Women and girls represent around 50% of Venezuelan migration to Brazil.^5^ Evidence shows that displaced women and girls face higher levels of gender violence, including rape and trafficking, in addition to other risks related to poverty, stigma, discrimination, language barriers and social exclusion, including deficient access to public services.^6^, ^7^, ^8^, ^9^ This poses significant risks to their health, particularly in domains such as sexual and reproductive health, and consequently jeopardises the prospects for equitable and sustainable development.^10^^,^^11^

We examine the healthcare access conditions, quality of care received, and financial risk protection in health among displaced Venezuelan women in the Brazilian cities of Boa Vista (Roraima state capital) and Manaus (Amazonas state capital). The analysis relies on novel primary survey data collected in 2021 for Venezuelan migrants and compares it with representative data for Brazilian women living in these cities, obtained from secondary health and expenditure surveys.

Our study considers forced displacement as a social determinant of health, which limits individual choices, and influences the relationships between health status and factors like socioeconomic position, gender and ethnicity.^12^ A core motivation for our study is to understand the relationship between forced displacement and progress towards Universal Health Coverage (UHC). UHC requires that health systems ensure equitable access to quality services for all those who need healthcare, without suffering financial hardship.^13^ Brazil has a national tax-funded health system, *Sistema Único de Saúde* (SUS), that should offer universal coverage to all residents.^14^ Yet obtaining timely access to public healthcare can be more complex in the most deprived regions, like Roraima, where local health systems have historically struggled to meet healthcare demands.^15^

There is a large body of literature on the effects of voluntary migration, but much less is known about the impacts of forced migration to escape continued deprivation and violence. The health challenges imposed by forced displacement have been studied previously in some South–South migration contexts, including in Latin America.^16^ A few studies have focused on women and girls migrating from countries in the south of Asia and in Sub-Saharan Africa to Europe and Australia.^7^^,^^17^^,^^18^ Recent research has highlighted important issues for Venezuelan migrant women in Brazil, including deficient access to maternal care,^19^ unmet family planning^19^, ^20^, ^21^, ^22^ and menstrual hygiene management needs,^22^^,^^23^ lack of information on access to sexual and reproductive health services,^20^^,^^22^ and risk of violence at migrant shelters.^24^ These studies offer valuable insights about key barriers to adequate healthcare encountered by migrant women, yet important methodological challenges—pertaining mainly to the unavailability of data for displaced groups, or of an adequate comparison groups for displaced individuals—make it difficult to draw firm conclusions from findings.^25^

We contribute to filling such gaps by investigating the associations between migrant status and healthcare access, quality and financial protection in health, comparing Venezuelan migrants and Brazilian women for different periods of time after migration.

## Methods

### Data sources

The *Redressing Gendered Health Inequalities of Displaced Women and Girls in contexts of Protracted Crisis in Central and South America* (ReGHID) survey was conducted in July–September 2021, collecting data from Venezuelan migrant women aged 15–49 who had been living in the cities of Boa Vista or Manaus in Brazil for up to three years by the time of the interview. The full sample includes 2012 women, 1257 from Boa Vista and 755 from Manaus. The survey collected socio-demographic data (e.g. education; household expenditure), health-related information (e.g. healthcare seeking, use and expenditures; quality of care received) and information about the migration journey. The sampling strategy was a Respondent Driven Sampling (RDS). This approach selects a population based on successive recruitment cycles, in which the probability of selection depends on the network size of the participant. Our final sample once adjusted for sampling statistical weights is representative for the Venezuelan migrant women who arrived in Brazil between 2018 and 2021. Further details about the sampling strategy and information collected are provided in the Supplementary Methods 1.

Data for comparable samples of Brazilian women come from two household surveys undertaken by the Brazilian Institute of Geography and Statistics (IGBE): the Brazilian Household Budget Survey, *Pesquisa de Orçamentos Familiares* (POF) 2017–2018,^26^ and the Brazilian National Health Survey, *Pesquisa Nacional de Saúde* (PNS) 2019.^27^ The PNS 2019 collected socio-demographic and health-related information for 94,414 households, including healthcare access and payments. The POF survey collected data for 57,920 households including monthly total household expenditures and healthcare expenditures. The POF and PNS data are representative of the general Brazilian population, and of urban populations at the state and capital city levels. Our ReGHID survey questionnaire was applied in urban settings and was designed to contain questions that are directly comparable (regarding phrasing and periods of reference) to the relevant POF and PNS questions. All interviewees signed an informed consent form before starting the interviews.

### Variable description

#### Populations

The key population of interest are Venezuelan women between the ages of 15–49 who had been living in Brazil for up to three years, in Boa Vista (Roraima state capital) or Manaus (Amazonas state capital), at interview time. The comparison population is comprised of Brazilian women of the same age group living in the same cities (in secondary analyses we use the total samples of Brazilian women living in the states of Roraima and Amazonas as an alternative comparison group, see Supplementary Methods 2).

#### Outcomes

We examine four groups of UHC-related outcomes: degree of financial risk protection in health, healthcare access in general, healthcare access in the specific domain of sexual and reproductive health (SRH) services, and quality of SRH services received. For all the analyses of financial protection outcomes, the data for Brazilian non-migrant women were taken from the Brazilian Household Budget Survey, whilst for all analyses of access and quality outcomes the data for Brazilian women come from the Brazilian National Health Survey. The Supplementary Methods 2 section presents the key survey questions used to construct all the indicators described below.

##### Financial risk protection

We measure financial risk protection through three indicators. The first is *out-of-pocket healthcare expenditures* (OOPHE), defined as all household payments incurred at the moment of receiving a health service. Health expenditures that take a high proportion of a household's total expenditure are defined as catastrophic health expenditures (CHE), an incidence measure widely used to track UHC progress.^13^ We use two alternative binary indicators of the *incidence of catastrophic health expenditures* in the household, defined as household healthcare expenditure amounting to at least 10% and 25% of total household expenditures (standard expenditure thresholds adopted for UHC monitoring indicators; e.g. Wagstaff et al.).^28^ Total household expenditure data refer to the total of products acquired and the services used by the household in a representative month. All household expenditure data are expressed in Brazilian *Reales* (R$) at 2021 values adjusting for inflation.

##### General healthcare access

Regarding general access to health services, we constructed a binary measure indicating whether a person *received care after actively seeking it*. Survey respondents were asked whether they sought any healthcare in the two weeks prior to interview; and if they did, whether or not they received it the first time they requested it. Additionally, we constructed a binary measure of *access to medications without payment in the health system*, based on a survey question for women who regularly take medications for any of four chronic conditions (hypertension, diabetes, asthma and depression), about the source of the last prescription and whether or not they made any payment for it.

##### Sexual and reproductive healthcare access

We examine access to sexual and reproductive health services through a binary indicator (constructed for women aged 25–64 as target population) of whether the woman had a *pap smear in the last three years*, as recommended by the Brazilian Cervical Cancer Screening Guidelines.^29^ Additionally, for women who were pregnant at interview time or gave birth in the year prior to the interview, we examine a binary indicator reflecting whether they *received at least one antenatal care consultation* during that pregnancy.

##### Quality of care

For quality of care, we constructed two binary indicators related to sexual and reproductive health. The first is an indicator of care timeliness that measures, for women aged 25–64 who had a pap smear in the previous three years, whether the woman *received the pap smear results in less than three months after the test*. Our second indicator assesses, for the group of women who received antenatal care consultations as explained above, whether such *antenatal care was delivered in accordance with the Brazilian guidelines*, by having blood, HIV and Hepatitis B tests conducted.^30^

#### Covariates

We use a range of socio-demographic categorical indicators to control for observable differences between Venezuelan migrant women and Brazilian women. All regression models include city of residence (Manaus indicator); age group (15–19, 20–29, 30–39, 40–49); education (less than primary education, primary, secondary education and higher education); ethnicity (mixed, white, black, indigenous, and other); and household size (number of family members living together). For non-financial protection outcomes, we can also control for income quintiles and marital status (married or lives with partner) from the Brazilian National Health Survey inflated to 2021 values.

### Matching methodology

We are interested in the differences in outcomes between migrant Venezuelan women and non-migrant Brazilian women. Because the observed characteristics may differ between migrant and local populations, we reweight the local population (comparison sample) to balance the distribution of covariates across both migrant and non-migrant populations. We use entropy balancing, an approach that allows us to re-weight the data to satisfy moment conditions in the covariate distribution.^31^ Through this approach we re-weight each observation in the Brazilian local population, leading to covariate distributions in the migrant and non-migrant samples that are fully balanced in their means, variances and skewness, thus enhancing population comparability for estimation purposes.

### Statistical analysis

We explore the association between being a migrant (i.e. migrant status) and our outcomes. Our estimates represent the probability of incurring catastrophic expenditures or the probability of accessing a given service associated with being a migrant; and the difference in the amount spent in healthcare associated with being a migrant compared to the local population. Using the weights derived from entropy balancing, for each outcome we estimate two versions of the weighted regression defined in equation (1):

(1) $$y_{ci}=\alpha_{0}+\beta M_{i}+\gamma M_{i}\times C_{i}+\delta C_{i}+\theta_{x}X_{i}+\varepsilon_{ci}$$

where *y*_*ci*_ refers to outcome *y* for woman *i* living in city *c*; *α*_*0*_ is the intercept; *M*_*i*_ is an indicator for whether the woman *i* is a migrant or not (migrant = 1); *C*_*i*_ is binary variable for living in Manaus or Boa Vista (Manaus = 1); **X**_*i*_ is a vector of socio-demographic controls at the household and individual level; and *ε*_*i*_ is the idiosyncratic error assumed to be uncorrelated to *y*. We also estimate a second (augmented) version of this equation for each outcome, where we add the interaction term *M*_*i*_ *x C*_*i*_ indicating whether *i* is a migrant woman living in Manaus, to investigate possible differences in the associations between migrant status and outcomes that depend on the place of settlement. We may expect some differences to exist because Boa Vista is the first large place of settlement of Venezuelans arriving in Northern Brazil (after arriving in the small border town of Pacaraima), while relatively wealthier Manaus represents a migration destination 780 km farther away, requiring extra time and resources to be reached by migrants.

We also estimate equation (2) to explore the variations in migrant outcomes associated with differences in time since arrival in Brazil. These estimates represent differences in the probability of incurring catastrophic expenditures in health or accessing healthcare services associated with time since arrival compared to local population; and the association of differences in the amount of healthcare spending with time since migrating.

(2) $$y_{ci}=\alpha_{0}\;+\;\sum_{t}^{T}\pi_{t}D_{ci}\;+\;\delta C_{i}\;+\;\theta_{x}X_{i}\;+\;\varepsilon_{ci}$$

where *D*_*ci*_ indicates the time since arrival in Brazil, measured in *T = 4* different categories: *t = 0* indicates non-migrant; *t = 1* less than 6 months living in Brazil; *t = 2* between 7 and 18 months since migrating; and *t = 3* more than 18 months. We add controls for city of residence and socio-demographic characteristics as in equation (1).

The reporting of this study conforms to the STROBE statement. The project was approved by the Research Ethics Committee of the Federal University of Maranhão, under protocol number: 35617020.9.1001.5087.

### Role of the funding source

The funder (Economic and Social Research Council-ESRC UK) had no involvement in the study design; collection, analysis and interpretation of data; writing of the paper; or in the decision to submit the paper for publication.

## Results

### Sample description

Table 1 shows the distribution of covariates and outcomes for migrants and non-migrants before entropy balancing. Around 39.3% (785/1997) of migrants reported having been living in Brazil for less than six months; 24.8% (496/1997) between seven and 18 months; and 35.9% (716/1997) more than 18 months. While 62.5% (1257/2012) of migrant women lived in Boa Vista and 37.5% (755/2012) in Manaus, most non-migrants in both the Brazilian Household Budget and the National Health surveys were Manaus residents.

**Table 1:** Descriptive statistics for migrants (ReGHID survey) and non-migrants (PNS and POF surveys).

	Migrants		Non-migrants			
	ReGHID		PNS		POF	
Months since migration (N (%))						
≤ 6	785/1997	(39.3)				
7–18	496/1997	(24.8)				
19–36	716/1997	(35.9)				
Age (N Mean (SD)), years	2012 29.46	(8.9)	3047 30.98	(9.9)	1649 30.51	(9.8)
Age group (N (%))						
<20	268/2012	(13.3)	494/3047	(16.2)	291/1649	(17.6)
20–29	845/2012	(42.0)	925/3047	(30.4)	493/1649	(29.9)
30–39	564/2012	(28.0)	879/3047	(28.8)	494/1649	(30.0)
40+	335/2012	(16.7)	749/3047	(24.6)	371/1649	(22.5)
Ethnicity (N (%))						
White	577/2011	(28.7)	694/3047	(22.8)	369/1644	(22.4)
Mixed	1286/2011	(63.9)	2068/3047	(67.9)	1140/1644	(69.3)
Black	71/2011	(3.5)	196/3047	(6.4)	81/1644	(4.9)
Indigenous	62/2011	(3.1)	70/3047	(2.3)	32/1644	(1.9)
Other	15/2011	(0.7)	19/3047	(0.6)	22/1644	(1.3)
Education (N (%))						
Less than primary	23/2011	(1.1)	10/2240	(0.4)	64/1649	(3.9)
Primary	293/2011	(14.6)	358/2240	(16.0)	517/1649	(31.4)
Secondary	1383/2011	(68.8)	1229/2240	(54.9)	673/1649	(40.8)
Higher	312/2011	(15.5)	643/2240	(28.7)	395/1649	(24.0)
Lives with partner (N (%))	1146/2011	(57.0)	1538/3047	(50.5)		
Household size (N Mean (SD))	2012 2.61	(2.3)	3040 4.3	(2.0)	1649 4.44	(1.9)
Household income (N (%)), R$						
No income	794/1885	(42.1)	43/3040	(1.4)		
<500	324/1885	(17.2)	88/3040	(2.9)		
500–1000	420/1885	(22.3)	173/3040	(5.7)		
>1000	345/1885	(18.3)	2736/3040	(90.0)		
City of residence (N (%))						
Boa Vista	1257/2012	(62.5)	1442/3047	(47.3)	591/1649	(35.8)
Manaus	755/2012	(37.5)	1605/3047	(52.7)	1058/1649	(64.2)
CHE 10% (N (%))	278/1802	(15.4)			81/1644	(4.9)
CHE 25% (N (%))	130/1802	(7.2)			10/1644	(0.6)
Health expenditure (N Mean (SD)), R$	1953 44.06	(154.3)			1644,116.6	(187.7)
Health outcomes (N (%))						
Received care when sought	638/670	(95.2)	392/564	(69.5)		
Did not pay for medications	92/143	(64.3)	33/68	(48.5)		
Good health	1425/2009	(70.9)	2610/3047	(85.7)		
Pap smear in last 3 years	465/947	(49.1)	725/847	(85.6)		
Antenatal care consultation	281/311	(90.4)	85/93	(91.4)		
Timely pap smear results	385/453	(85.0)	625/717	(87.2)		
Antenatal care as per guidelines	236/267	(88.4)	71/93	(76.3)		
Sexual transmitted infections	16/2012	(0.8)	12/1085	(1.1)		

POF: data from the Brazilian Household Budget Survey (*Pesquisa de Orçamentos Familiares*).

PNS: data from the Brazilian National Health Survey (*Pesquisa Nacional de Saúde*).

ReGHID: data from the ReGHID survey (Redressing Gendered Health Inequalities of Displaced Women and Girls in contexts of Protracted Crisis in Central and South America).

CHE: catastrophic health expenditures (healthcare expenditures exceed 10% or 25% of total household expenditure).

R$: Brazilian Real.

Among migrant women, 15.4% (278/1802) and 7.2% (130/1802) incurred catastrophic health spending at the 10% and 25% household expenditure thresholds (respectively), compared to only 4.9% (81/1644) and 0.6% (10/1644) (respectively) among non-migrants. Mean household out-of-pocket health expenditure was R$44.06 (S.D. 154.3) for migrant women and R$116.6 (S.D. 187.7) for non-migrants. Most migrant women received healthcare when sought (95.2% = 638/670); this proportion was lower for non-migrants (69.5% = 392/564). The migrant sample also had higher proportions of women receiving their medication without payment (64.3% = 92/143 vs. 48.5% = 33/68) and antenatal care according to guidelines (88.4% = 236/267 vs. 76.3% = 71/93) compared to the non-migrant sample. By contrast, the migrant sample displayed lower proportions of women with a pap smear in the last three years (49.1% = 465/947 vs. 85.6% = 725/847), timely pap smear test results (85.0% = 385/453 vs. 87.2% = 625/717) and an antenatal care consultation in their last pregnancy (90.4% = 281/311 vs. 91.4% = 85/93) than the local comparisons.

There were important differences between the migrant and non-migrant samples in a few socio-demographic characteristics. For example, 42.1% (794/1885) of migrants reported no income and only 18.3% (345/1885) had an income over R$1000, compared to non-migrant figures of 1.4% (43/3040) and 90% (2736/3040) respectively for non-migrants. Lower proportions of migrant women had less than primary (1.1% = 23/2011) or up to primary education (14.6% = 293/2011) compared to non-migrants in the POF sample (3.9% = 64/1649, and 31.4% = 517/1649, respectively). Applying our entropy balancing weighting approach resulted in distributions of covariates that are virtually identical between the migrant and non-migrant samples (e.g. differences in mean values of one percentage point or less, and statistically insignificant; see Supplementary Table S1- for descriptive statistics after matching). The differences in the numbers of observation across variables are due to data availability. The analyses were conducted with the data available.

### Regression results

Tables 2 and 3 show the associations between outcomes and migrant status, obtained by estimating equation (1) via ordinary least squares with entropy balancing. Tables 4 and 5 display regression results from estimating equation (2) through the same approach, according to time since migration. We explore differences in outcomes depending on time since migration by testing the hypotheses of 1) whether the association coefficients estimated for migrants who have been living for 6 months or less in Brazil are statistically different from the corresponding coefficients for those migrants who have been living in Brazil for any longer period (d1 = d2, d1 = d3); and 2) whether the coefficients for migrants living in Brazil for 7–18 months are different from the corresponding coefficients for those who have been living in Brazil for more than 18 months (d2 = d3). We display the resulting F-statistics and p-values for both tests in Tables 4 and 5.

**Table 2:** Financial protection regression results.

	Financial protection					
	Catastrophic health expenditure 10%		Catastrophic health expenditure 25%		Health Expenditure (R$)	
	(Model 1)	(Model 2)	(Model 3)	(Model 4)	(Model 5)	(Model 6)
Migrant	0.095 [0.06–0.13]	0.006 [−0.04 to 0.06]	0.066 [0.05–0.08]	0.051 [0.03–0.07]	−46.48 [−65.2 to −27.76]	−71.08 [−96.59 to −45.57]
	(<0.0001)	(0.82)	(<0.0001)	(<0.0001)	(<0.0001)	(<0.0001)
Migrant∗Manaus		0.246 [0.18–0.31]		0.041 [0.01–0.07]		66.52 [33.49–99.56]
		(<0.0001)		(0.0009)		(0.0001)
Manaus	0.067 [0.03–0.10]	−0.047 [−0.10 to 0.01]	0.019 [0.01–0.04]	−0.000 [−0.01 to 0.01]	31.21 [10.76–51.67]	−1.14 [−33.71 to 31.44]
	(<0.0001)	(0.09)	(0.009)	(0.96)	(0.003)	(0.95)
Observations	3439	3439	3439	3439	3590	3590
R-squared	0.045	0.082	0.038	0.040	0.086	0.096
F-stat	8.33	12.38	9.523	9.074	8.691	11.22
Prob > F	<0.0001	<0.0001	<0.0001	<0.0001	<0.0001	<0.0001

Robust p values in parentheses.

95% CI in brackets.

R$: Brazilian Real.

Migrant∗Manaus is the interaction term of migrant and Manaus. Refers to migrants living in the city of Manaus.

Models 1 to 4 present the probabilities of incurring catastrophic health expenditures (10% or 25%) associated with being a migrant (vs. local), being a migrant in Manaus (vs. migrant in Boa Vista) or living in Manaus (vs. living in Boa Vista). Models 5–6 show the difference in healthcare expenditures in R$ associated with being a migrant in Manaus, and living in Manaus (migrant and non-migrant). All models are adjusted for sociodemographic characteristics of women.

F-stat: Tests whether there is no predictive relationship between the regressors and the outcome in the models. We reject the null hypothesis of no predictive relationship with values of Prob > F smaller than 0.05.

**Table 3:** Healthcare access and quality results.

	Healthcare access and quality			
	General health			
	Access to care			
	Received care when sought		Did not pay for medications	
	(Model 1)	(Model 2)	(Model 3)	(Model 4)
Migrant	0.270 [0.11–0.43]	0.296 [0.08–0.51]	0.124 [−0.08 to 0.33]	0.060 [−0.21 to 0.33]
	(0.0008)	(0.008)	(0.23)	(0.66)
Migrant∗Manaus		−0.075 [−0.40 to 0.25]		0.147 [−0.18 to 0.47]
		(0.65)		(0.37)
Manaus	−0.051 [−0.19 to 0.09]	−0.011 [−0.31 to 0.29]	−0.530 [−0.69 to −0.37]	−0.628 [−0.89 to −0.36]
	(0.47)	(0.94)	(<0.0001)	(<0.0001)
Observations	1–053	1–053	184	184
R-squared	0.453	0.454	0.438	0.441
F-stat	1.313	1.582	16.10	21.79
Prob > F	0.18	0.06	<0.0001	<0.0001

	Sexual and reproductive health			
	Access to care		Quality of care	
	Pap smear in the last 3 years		Timely pap smear results	
	(Model 5)	(Model 6)	(Model 7)	(Model 8)
Migrant	−0.368 [−0.45 to −0.29]	−0.437 [−0.55 to −0.33]	0.068 [−0.09 to 0.22]	0.056 [−0.14 to 0.25]
	(<0.0001)	(<0.0001)	(0.39)	(0.56)
Migrant∗Manaus		0.162 [−0.01 to 0.34]		0.024 [−0.22 to 0.27]
		(0.07)		(0.84)
Manaus	0.104 [0.02–0.19]	0.022 [−0.13 to 0.17]	−0.009 [−0.16 to 0.14]	−0.020 [−0.25 to 0.21]
	(0.017)	(0.77)	(0.90)	(0.87)
Observations	1–615	1–615	1–039	1–039
R-squared	0.211	0.216	0.078	0.078
F-stat	24.63	25.86	3.285	3.071
Prob > F	<0.0001	<0.0001	<0.0001	<0.0001

	Antenatal care consultation		Antenatal care as per guidelines	
	(Model 9)	(Model 10)	(Model 11)	(Model 12)
Migrant	0.141 [−0.11 to 0.39]	0.358 [−0.02 to 0.74]	0.148 [−0.10 to 0.40]	0.384 [0.01–0.76]
	(0.26)	(0.07)	(0.23)	(0.046)
Migrant∗Manaus		−0.507 [−0.96 to −0.06]		−0.553 [−1.00 to −0.11]
		(0.027)		(0.016)
Manaus	0.153 [0.02–0.29]	0.449 [0.09–0.81]	0.181 [0.03–0.33]	0.493 [0.13–0.85]
	(0.028)	(0.014)	(0.022)	(0.007)
Observations	374	374	331	331
R-squared	0.224	0.301	0.209	0.290
F-stat	1.512	1.889	2.265	2.446
Prob > F	0.093	0.018	0.004	0.001

Robust p values in parentheses.

95% CI in brackets.

Migrant∗Manaus is the interaction term of migrant and Manaus. Refers to migrants living in the city of Manaus.

This table presents the probabilities of receiving each health service associated with being a migrant (vs local), being a migrant in Manaus (vs. migrant in Boa Vista) or living in Manaus (vs. living in Boa Vista).

All models are adjusted for sociodemographic characteristics of women.

F-stat: Tests whether there is no predictive relationship between the regressors and the outcome in the models. We reject the null hypothesis of no predictive relationship with values of Prob > F smaller than 0.05.

**Table 4:** Financial protection results according to time since migration.

	Financial protection		
	Catastrophic exp 10%	Catastrophic exp 25%	Health expenditures R$
	(Model 1)	(Model 2)	(Model 3)
Time in Brazil			
Less than 6 months	0.020 [−0.02 to 0.06]	0.046 [0.03–0.06]	−66.02 [−86.48 to −45.55]
	(0.37)	(<0.0001)	(<0.0001)
Between 7 and 18 months	0.118 [0.07–0.16]	0.082 [0.05–0.11]	−50.80 [−71.22 to −30.38]
	(<0.0001)	(<0.0001)	(<0.0001)
Between 19 and 36 months	0.159 [0.12–0.20]	0.078 [0.06–0.10]	−22.13 [−44.45 to −0.20]
	(<0.0001)	(<0.0001)	(0.05)
Manaus	0.041 [0.00–0.08]	0.013 [0.00–0.03]	23.82 [2.70–44.95]
	(0.033)	(0.07)	(0.027)
Observations	3427	3427	3578
R-squared	0.062	0.041	0.092
F-stat	9.674	8.316	9.658
Prob > F	<0.0001	<0.0001	<0.0001
F-stat d1 = d2 d1 = d3	26.92	3.993	11.34
p > F d1 = d2 d1 = d3	<0.0001	0.019	<0.0001
F-stat d2 = d3	2.673	0.032	8.686
p > F d2 = d3	0.10	0.86	0.003

Robust p values in parentheses.

95% CI in brackets.

Models 1 and 2 present the probabilities of incurring catastrophic health expenditures (10% or 25%) associated with the time since arriving to Brazil: less than 6 months between 7 and 18 months, and between 19 and 36 months. Model 3 shows the difference in healthcare expenditures in R$ associated with time since arriving to Brazil.

All models are adjusted for sociodemographic characteristics of women.

F-stat: Tests whether there is no predictive relationship between the regressors and the outcome in our model. We reject the null hypothesis of no predictive relationship with values of Prob > F under 0.05.

p > F d1 = d2 d1 = d3 tests whether being in Brazil for 6 months or less (d1) is statistically different than being for more than 6 months (d2 & d3) for each outcome.

p > F d2 = d3 tests whether being in Brazil for 7–18 months (d2) is statistically different than being for 19–36 months for each outcome.

**Table 5:** Healthcare access and quality results according to time since migration.

	Healthcare access and quality					
	General health		Sexual and reproductive health			
	Received care when sought	Did not pay for medications	Pap smear in last three years	Antenatal care consultation	Timely pap smear results	Antenatal care as per guidelines
	(Model 1)	(Model 2)	(Model 3)	(Model 4)	(Model 5)	(Model 6)
**Months in Brazil**						
Less than 6	0.278 [0.10–0.46]	0.090 [−0.14 to 0.32]	−0.379 [−0.48 to −0.28]	0.112 [−0.21 to 0.44]	0.097 [−0.07 to 0.27]	0.162 [−0.17 to 0.50]
	(0.002)	(0.44)	(<0.0001)	(0.50)	(0.27)	(0.34)
Between 7 and 18	0.268 [0.11–0.43]	0.147 [−0.13 to 0.42]	−0.320 [−0.42 to −0.22]	0.189 [−0.07 to 0.45]	0.078 [−0.09 to 0.25]	0.147 [−0.13 to 0.42]
	(0.001)	(0.29)	(<0.0001)	(0.16)	(0.36)	(0.29)
Between 19 and 36	0.255 [0.11–0.40]	0.148 [−0.08 to 0.38]	−0.386 [−0.47 to −0.30]	0.132 [−0.07 to 0.33]	0.040 [−0.12 to 0.20]	0.142 [−0.06 to 0.35]
	(0.001)	(0.21)	(<0.0001)	(0.20)	(0.62)	(0.18)
Manaus	−0.048 [−0.19 to 0.10]	−0.524 [−0.70 to −0.35]	0.101 [0.01–0.19]	0.151 [−0.00 to 0.30]	−0.002 [−0.15 to 0.15]	0.183 [0.02–0.35]
	(0.52)	(<0.0001)	(0.023)	(0.05)	(0.98)	(0.032)
Observations	1049	182	1607	374	1034	331
R-squared	0.454	0.437	0.213	0.229	0.079	0.210
F-stat	0.183	13.67	21.89	1.863	2.835	2.028
Prob > F	0.67	<0.0001	<0.0001	0.018	<0.0001	0.008
F-stat d1 = d2 d1 = d3	0.150	0.165	1.292	1.227	0.550	0.023
p > F d1 = d2 d1 = d3	0.86	0.85	0.28	0.30	0.58	0.98
F-stat d2 = d3	0.183	0.000	2.203	1.202	0.606	0.007
p > F d2 = d3	0.67	0.99	0.14	0.27	0.44	0.94

Robust p values in parentheses.

95% CI in brackets.

This table presents the probabilities of receiving each health service associated with time since arriving to Brazil: less than 6 months; between 7 and 18 months; and, between 19 and 36.

All models are adjusted for sociodemographic characteristics of women.

F-stat: Tests whether there is no predictive relationship between the regressors and the outcome in our model. We reject the null hypothesis of no predictive relationship with values of Prob > F under 0.05.

p > F d1 = d2 d1 = d3 tests whether being in Brazil for 6 months or less (d1) is statistically different than being for more than 6 months (d2 & d3) for each outcome.

p > F d2 = d3 tests whether being in Brazil for 7–18 months (d2) is statistically different than being for 19–36 months for each outcome.

#### Financial risk protection

We find that being a migrant is associated with lower household out-of-pocket health spending, by R$46.48 (95% CI: −65.2 to −27.8; p < 0.0001) (Table 2, model 5). This lower spending among migrant women is particularly driven by migrants in Boa Vista: conditional on being a migrant, women in Manaus exhibit higher out-of-pocket health spending than those in Boa Vista, by R$66.52 (95% CI: 33.5–99.6; p = 0.0001) (Table 2, model 6). However, from Table 4, we see that the health spending difference between migrants and non-migrants tends to decrease according to time since settlement in Brazil (Table 4, model 3). Whilst arrival in the last 6 months is associated with R$66.02 (95% CI: −86.5 to −45.6; p < 0.0001) lower spending for migrants compared to non-migrants, this difference decreases to R$50.80 (95% CI: −71.2 to −30.4; p < 0.0001) at least for longer periods since arrival in Brazil.

At the 10% household expenditure threshold, migrant women had a higher incidence of catastrophic health expenditures (CHE) than non-migrant women, by 9.5 percentage points (pp) (95% CI: 6pp–13pp; p < 0.0001) (Table 2, model 1). This difference was driven by a much higher CHE incidence for migrants in Manaus than in Boa Vista (by 24.6 pp; 95% CI: 18–31; p < 0.0001; Table 2, model 2). The mean difference in CHE incidence between migrant and non-migrant women is statistically significant for those migrants with more than 6 months of residence in Brazil, increasing by at least 11.8 pp (p < 0.0001) (Table 4, model 1). A very similar picture emerges from the estimations for catastrophic expenditures considering the 25% expenditure threshold, with higher catastrophic expenditure incidence among migrant women (6.6 pp; 95% CI: 5 pp–8 pp; p < 0.0001) that was driven by migrants in Manaus (4.1 pp; 95% CI: 1 pp–7 pp; p = 0.0009) (Table 2, models 3–4), and a higher mean incidence rate for those migrants with more than 6 months in Brazil (p < 0.0001) (Table 4, model 2).

#### General healthcare access

We find that migrant women were more likely than non-migrants to have received healthcare when sought by 27 percentage points (95% CI: 11 pp–43 pp; p = 0.0008), with no statistically significant differences according to city of residence (Table 3, models 1–2). This difference in general healthcare access favouring migrant women was apparent regardless of time since migration (Table 5, model 1). There was no difference between migrants and non-migrants in the likelihood of receiving chronic care medications for free (Table 3, model 3), irrespective of city of settlement (Table 3, model 4) or time since migration (Table 5, model 2).

#### Sexual and reproductive healthcare access

Migrants were 36.8 percentage points (95% CI: −45 pp to −29 pp; p < 0.0001) less likely than non-migrants to have had a pap smear in the last 3 years (Table 3, model 5), a difference that persisted regardless of time since migration (Table 5, model 3). We also find no statistically significant difference between migrant and non-migrant women in having received at least one antenatal consultation during pregnancy (Table 3, model 9), irrespective of time since migration (Table 5, model 4).

#### Quality of care

Among women who had a pap smear, migrants were as likely as non-migrants to have received their test results in less than three months (Table 3, model 7). Similarly, among women who received an antenatal consultation, migrants were as likely as non-migrants to have received consultations in accordance with clinical guidelines (Table 3, model 11), irrespective of time living in Brazil (Table 5, model 6).

We conducted a variance factor analysis (VIF) to check for multicollinearity in our covariates which strongly suggest that our main results are unaffected by multicollinearity (see Supplementary Methods 3).

#### Sensitivity analyses

We present the full results for the regressions above, with the estimated coefficients for all covariates, in the Supplementary Material (Supplementay Tables S3–S7). Finally, we conducted three sensitivity analyses: estimating the regressions using a logit procedure instead of linear regressions for binary outcomes (Supplementay Tables S11–S13); changing the comparison samples to include all Brazilian women living in the states of Roraima and Amazonas (Supplementay Tables S8–S10); and estimating the models accounting for survey sample weights (Supplementay Tables S14–S16). These additional estimations led to conclusions that were largely unchanged, both in terms of the direction and magnitude of associations.

## Discussion

Progress towards Universal Health Coverage requires that health systems ensure equitable access to services with financial protection for all—including displaced populations settling in a new country. Our analysis of two populations of displaced Venezuelan women and adolescent girls, in the Brazilian cities of Boa Vista and Manaus, paints a mixed picture. Venezuelan migrant women had adequate healthcare access and quality compared to their Brazilian counterparts. This is reassuring in the context of very high vulnerability among displaced Venezuelan women facing a traumatic-life event likely to produce short- and long-term effects on health due to stress,^32^ poor integration in host communities including deficient access to public and quality healthcare,^33^ precarious life arrangements prior to resettlement (i.e. poor sanitary conditions, inadequate shelter, crowding and suboptimal nutrition),^34^ and exposure to different types of violence,^24^^,^^35^ among others. This situation is corroborated by our data, e.g. by generally worse self-assessed health among migrant women than for Brazilian women (Supplementary Table S4).

Yet our data reveal inadequate standards of financial risk protection in health among migrant women, which has the potential to widen gender gaps and reinforce poverty. Our finding that Venezuelan women in the cities of Boa Vista and Manaus have lower levels of household out-of-pocket expenditure in health than comparable Brazilian women is hardly surprising. This highlights the tight budget constraints and tough spending choices that migrant women face when the need for healthcare arises. This difference between migrants and non-migrants is driven by migrant women living in Boa Vista, who spend significantly less in healthcare than those in Manaus. This may reflect the generally better socioeconomic status of Manaus migrants, who may then be better able to afford to spend privately on healthcare needs (e.g. prescription medicines that they have been unable to obtain from the public system) than their Boa Vista counterparts and may hint at unmet healthcare needs among the latter. The differences in healthcare spending between migrant and Brazilian women tend to decrease with time since migration, supporting a view that longer assimilation time in the host communities contributes to bring migrants closer to the healthcare expenditure patterns displayed by the native population.

Despite the lower average healthcare spending by migrant Venezuelan women, there is a higher incidence of catastrophic healthcare expenditure among the latter, compared to Brazilian women. This raises questions about why the health system in Brazil—a country where publicly funded universal healthcare is enshrined in its Constitution—seems to be facing challenges to protect displaced women from financially catastrophic healthcare payments. The higher incidence of catastrophic expenditures among migrants is driven by women settled in Manaus. The latter also tend to be those with more time since migration compared to migrants in Boa Vista, which is reflected in higher catastrophic expenditures incidence for those with more than six months since migration. The fact that Manaus migrant women have, on average, higher out-of-pocket healthcare expenditures than their Boa Vista counterparts, is likely to be a key explanatory factor of the higher catastrophic health expenditures incidence among Manaus migrants. There is a large body of literature documenting the strong positive correlation between out-of-pocket health expenditures in a health system and the locally observed incidence of catastrophic and impoverishing healthcare spending.^28^

Perhaps surprisingly, we find that Venezuelan migrant women in both Boa Vista and Manaus have better access to general healthcare when they seek care, compared to non-migrants, and for any period of time since migration. Migrant women also have the same likelihood of obtaining their chronic care medicines cost-free in the Brazilian health system. These relatively good access conditions for migrant women align with findings about the Brazilian health system from other studies.^9^ For “recent” migrants (i.e. those who had arrived in Brazil closer to the time of our survey), part of the good access conditions may reflect the proactive signposting to basic health services and public facilities, provided by Brazilian officials, religious organisations and international organisation representatives (e.g., United Nations High Commissioner for Refugees) at the migrant shelters.^14^ Yet some caution is warranted in interpreting our general access results. First, our general healthcare access indicator refers to care received when sought, so it does not capture situations where women have health needs but decide not to seek care, for instance due to lack of information, capacity to recognise health-related risk, transportation costs, among other causes. Second, our findings about access to free medications are specific for the limited set of four chronic conditions included in our indicator. The higher incidence rate of catastrophic healthcare expenditures among migrants suggests that Venezuelan migrant women may still need to pay privately for medicines to treat other conditions, and/or have to purchase other health services from private providers in order to follow treatment pathways, possibly because of limited availability in the public system as identified in other settings.^36^ There is evidence from many countries indicating that prescription drugs and diagnostic tests often constitute major drivers of catastrophic payments.^37^ Although, in principle, the unavailability of certain drugs and tests in Boa Vista and Manaus public healthcare facilities could drive private payments by migrant and Brazilian women alike, these payments would probably lead to a higher risk of catastrophic expenditure among migrant women (as we find in our estimations), given their generally lower income levels.

For access specifically regarding sexual and reproductive health services, we found a nuanced picture. There is no evidence of systematic differences in access to antenatal care between migrants and non-migrants, which highlights the more general point that existing access gaps in the public health network affect migrant and Brazilian women equally for certain healthcare domains. The latter point is corroborated by persisting gaps in access to sexual and reproductive health services for Venezuelan migrants which have been identified not only in antenatal care, but also in other areas including postnatal care, family planning and menstrual products.^19^, ^20^, ^21^, ^22^ By contrast, we found that migrant women had a much lower likelihood than non-migrants of having undergone a pap smear in the last three years, a situation that persists at least up to three years since migration. This may reflect, partly at least, a backlog of unmet healthcare needs that these women have had since before their displacement from Venezuela. Further research is needed to understand the root causes of this discrepancy, which could include factors such as different levels of knowledge or acceptance of the test among Venezuelan women.

Concerning sexual and reproductive health services, compared to Brazilian women, migrants were as likely to have received pap smear test results in less than three months and as likely to have received antenatal care according to guidelines. Although these findings may partly reflect care obtained in Venezuela, the well-documented current fragility of the Venezuelan health system,^1^ along with the main reasons driving the migration decision revealed by the Venezuelan women interviewed (where difficulty of accessing healthcare was mentioned by 37.8%, representing the second most important concern leading to displacement, after difficulties to obtain food),^26^ suggests that migrant women have indeed seen improvements in sexual and reproductive health care quality indicators after migrating to Brazil. This adds to evidence indicating good levels of satisfaction in specific domains like obstetric care among Venezuelan migrant women living in Brazil.^21^

Our analyses, coupled with evidence discussed elsewhere,^12^ offer insights for the design of policy responses to mitigate harmful consequences of displacement among migrant Venezuelan women. Proactive initiatives by the Brazilian health system seem warranted to provide migrant women with more comprehensive information about the availability of health services in the places of arrival and settlement, and about how to seek those services, in all healthcare domains (including primary and secondary care). Currently, the information provided to migrant women is focused on services considered to be integral to a short-term emergency response, e.g. contraception, whilst there is insufficient knowledge among migrants about how to access other family planning and maternal health services.^20^, ^21^, ^22^ This may be leading to important gaps in knowledge among displaced women relating to how/where to obtain a wide range of other public health services, including services that may lead to high out-of-pocket expenditures should women end up purchasing them from private providers. From a healthcare supply perspective, the Brazilian universal healthcare system SUS must ensure appropriate mobilisation of financial resources to the local health systems hosting these displaced populations, to allow their adequate access to services ranging from health promotion and preventive care to diagnosis and treatment. A particularly key aspect is to ensure that the increased public provision of consultation/diagnostic services to Venezuelan migrants in Brazil (compared to their previous situation in Venezuela) is accompanied by adequate access to follow-up treatment services. Qualitative interviews suggest that some Venezuelan women may spend months and years waiting for specialised care in sexual and reproductive health and other domains.^12^ Delays in access may lead to a worsening of health conditions and more expensive treatment services later down the line, with gaps in public provision potentially leading to high out-of-pocket healthcare spending by these migrants.

From the perspective of displacement as a social determinant of health, our work also emphasises the importance, for migrant health, of social policies implemented beyond the health system.^12^^,^^38^ The findings here and elsewhere^20^^,^^21^ confirm that certain structural factors, such as potentially impoverishing healthcare costs, and informational or linguistic barriers for taking advantage of publicly provided services, tend to affect migrants disproportionately. This implies that the scope of public actions to support migrant health must be expanded towards ensuring that migrants have adequate information about and access to economic opportunities, social welfare and anti-poverty initiatives, among others.

Our study has limitations. We are constrained by having information on only a few proxy indicators for each Universal Healthcare Coverage objective.^13^ For example, quality of care is a wide-ranging concept that includes both objective and patient-subjective domains, which go beyond what we can examine in this study. Detailed information about factors like access to pregnancy termination for medical reasons or sexually transmitted infection testing would have allowed a more rounded assessment of sexual and reproductive care access, complementing our analyses of antenatal care and pap smear coverage. Moreover, it would have been useful to understand how migrants' healthcare spending is distributed across categories (direct and indirect) and services (e.g. sexual and reproductive services), yet our dataset captures only the total amount spent directly on medical goods and services, not covering indirect spending like travel costs to seek treatment, which may be important.^36^ Transportation costs may also act as healthcare seeking barriers that could influence care seeking patterns differently between migrant and Brazilian women, potentially skewing our comparative assessment of utilisation indicators and healthcare spending. We do not have information either about migrants’ service use patterns across public or private providers, which would have allowed further exploration of our hypotheses about catastrophic spending drivers. Future data collection efforts are needed to address these issues. Finally, we note that our cross-sectional estimates must be interpreted as associations in the data, rather than causal relationships. We cannot rule out the influence of unobserved confounders that may be correlated with migrant status and affect the relationships of interest. Future research could rely on appropriate empirical strategies, such as instrumental variable estimation using credible instruments within a natural experiment setting, to ascertain the causal links between migration and universal healthcare coverage-related indicators, in the Venezuela–Brazil corridor and other contexts.^39^

## Contributors

Moreno-Serra R: Conceptualisation, methodology, writing original draft, review and editing.

Ochoa-Moreno I: Formal analysis, visualization, writing original draft, review and editing.

Anaya-Montes M: Data curation, investigation.

Cardoso Fernandes L: Data curation, investigation.

Gomes T: Investigation, data curation.

Leal MDC: Conceptualisation, investigation, data curation.

Cuadrado C: Conceptualisation, investigation, data curation.

## Data sharing statement

All data used in the analyses are available upon request.

## Declaration of interests

Luis Fernandes has relocated to Janssen Pharmaceutica N.V., Beerse, Belgium. This disclaimer is to clarify that at the time of study development, the author was affiliated with the Centre for Health Economics, University of York. However, the author's current affiliation at Janssen Pharmaceutica N.V. does not introduce any conflicts of interest regarding the research findings, data interpretation, or conclusions presented in the paper. The author remains committed to upholding scientific integrity and ensuring the objectivity and impartiality of the research.

## References

[1] Espana L.P.N., Ponce M.G.Z. (2017).

[2] Olivieri S., Ortega F., Rivadeneira A., Carranza E. (2022). The labour market effects of venezuelan migration in Ecuador. J Dev Stud.

[3] (2024). Polícia federal atualiza números da migração de venezuelanos em RR [Federal Police updates Venezuelan migration numbers in RR].

[4] da Frota Simões G. (2017). http://visas.blog.br/documentos/perfil-sociodemografico-laboral-venezuelano-brasil.pdf.

[5] International Organization for Migration Analysis (2024). https://data.unhcr.org/en/documents/details/66845.

[6] Barot S. (2017). https://www.guttmacher.org/gpr/2017/02/state-crisis-meeting-sexual-and-reproductive-health-needs-women-humanitarian-situations.

[7] Temin M., Montgomery M., Engebretsen S., Barker K. (2013). Girls on the move: adolescent girls & migration in the developing world. Poverty Gend Youth.

[8] Valdez E.S., Valdez L.A., Sabo S. (2015). Structural vulnerability among migrating women and children fleeing central America and Mexico: the public health impact of “Humanitarian Parole”. Front Public Health.

[9] Brizuela V., Bahamondes L., Gómez Ponce de León R. (2023). Strengthening locally led research to respond to the sexual and reproductive health and rights of migrants from Venezuela and central America. Rev Panam Salud Pública.

[10] Menjívar C., Walsh S.D. (2017). The architecture of feminicide: the state, inequalities, and everyday gender violence in Honduras. Lat Am Res Rev.

[11] Standing H., Hawkins K., Mills E., Theobald S., Undie C.-C. (2011). Introduction: contextualising “rights” in sexual and reproductive health. BMC Int Health Hum Rights.

[12] Castañeda H., Holmes S.M., Madrigal D.S., Young M.-E.D., Beyeler N., Quesada J. (2015). Immigration as a social determinant of health. Annu Rev Public Health.

[13] World Health Organization and World Bank (2023).

[14] Cintra N., Owen D., Riggirozzi P. (2023).

[15] Massuda A., Hone T., Leles F.A.G., de Castro M.C., Atun R. (2018). The Brazilian health system at crossroads: progress, crisis and resilience. BMJ Glob Health.

[16] Pan American Health Organization (2019). https://www.paho.org/en/node/62823.

[17] Chant S., Klett-Davies M., Ramalho J. (2017). https://eprints.lse.ac.uk/84297/1/Young%20Female%20Adolescents%20in%20Urban%20Areas%20RER%20FINAL.pdf.

[18] Metusela C., Ussher J., Perz J. (2017). “In My Culture, We Don't Know Anything about That”: sexual and reproductive health of migrant and refugee women. Int J Behav Med.

[19] Bahamondes L., Laporte M., Margatho D. (2020). Maternal health among Venezuelan women migrants at the border of Brazil. BMC Public Health.

[20] Soeiro R.E., Rocha L., Surita F.G., Bahamondes L., Costa M.L. (2022). A neglected population: sexual and reproductive issues among adolescent and young venezuelan migrant women at the northwestern border of Brazil. Int J Gynecol Obstet.

[21] Makuch M.Y., Osis M.J.D., Brasil C., de Amorim H.S.F., Bahamondes L. (2021). Reproductive health among Venezuelan migrant women at the northwestern border of Brazil: a qualitative study. J Migr Health.

[22] Rocha L., Soeiro R., Gomez N., Costa M.L., Surita F.G., Bahamondes L. (2022). Assessment of sexual and reproductive access and use of menstrual products among Venezuelan migrant adult women at the Brazilian–Venezuelan border. J Migr Health.

[23] Soeiro R.E., Rocha L., Surita F.G., Bahamondes L., Costa M.L. (2021). Period poverty: menstrual health hygiene issues among adolescent and young Venezuelan migrant women at the northwestern border of Brazil. Reprod Health.

[24] Makuch M.Y., Osis M.J.D., Becerra A., Brasil C., de Amorim H.S.F., Bahamondes L. (2021). Narratives of experiences of violence of Venezuelan migrant women sheltered at the northwestern Brazilian border. PLoS One.

[25] Spiegel P.B., Bennedsen A.R., Claass J. (2007). Prevalence of HIV infection in conflict-affected and displaced people in seven sub-Saharan African countries: a systematic review. Lancet.

[26] (2019). Pesquisa de orçamentos familiares 2017–2018: Primeiros resultados [family budget survey 2017–2018: first results].

[27] Stopa S.R., Szwarcwald C.L., Oliveira MM de (2020). Pesquisa Nacional de Saúde 2019: histórico, métodos e perspectivas [National Health Survey 2019: history, methods and perspectives]. Epidemiol E Serviços Saúde.

[28] Wagstaff A., Flores G., Hsu J. (2018). Progress on catastrophic health spending in 133 countries: a retrospective observational study. Lancet Glob Health.

[29] (2018). Diretrizes brasileiras para o rastreamento do câncer do colo do útero [Brazilian guidelines for cervical cancer screening].

[30] Atenção ao pré-natal de baixo risco/Ministério da Saúde [Low-risk prenatal care/Ministry of Health]. Ministério da Saúde. Secretaria de Atenção à Saúde (2012).

[31] Hainmueller J. (2012). Entropy balancing for causal effects: a multivariate reweighting method to produce balanced samples in observational studies. Polit Anal.

[32] Haukka J., Suvisaari J., Sarvimäki M., Martikainen P. (2017). The impact of forced migration on mortality: a cohort study of 242,075 finns from 1939–2010. Epidemiology.

[33] Bauer T.K., Giesecke M., Janisch L.M. (2018). The impact of forced migration on mortality: evidence from German pension insurance records. Demography.

[34] Shackelford B.B., Cronk R., Behnke N. (2020). Environmental health in forced displacement: a systematic scoping review of the emergency phase. Sci Total Environ.

[35] Mollica R.F., Sarajlić N., Chernoff M., Lavelle J., Vuković I.S., Massagli M.P. (2001). Longitudinal study of psychiatric symptoms, disability, mortality, and emigration among Bosnian refugees. JAMA.

[36] Xu K., Evans D.B., Kadama P. (2006). Understanding the impact of eliminating user fees: utilization and catastrophic health expenditures in Uganda. Soc Sci Med.

[37] Global monitoring report on financial protection in health 2021 (2021).

[38] Wickramage K., Vearey J., Zwi A.B., Robinson C., Knipper M. (2018). Migration and health: a global public health research priority. BMC Public Health.

[39] Cuadrado C., Libuy M., Moreno-Serra R. (2023). What is the impact of forced displacement on health? A scoping review. Health Policy Plan.

