# Supplementary material for: Healthcare access, quality and financial risk protection among displaced Venezuelan women living in Brazil: a cross-sectional study
Source: Lancet Reg Health Am. 2024 Jun 29;37:100830. doi: 10.1016/j.lana.2024.100830 (PMC11519684; doi:10.1016/j.lana.2024.100830)
Supplement: Supplementary material [file mmc1.pdf]

Supplementary material

Healthcare access, quality and financial risk protection among displaced Venezuelan women living in Brazil: A cross-sectional study

Rodrigo Moreno-Serra, Ivan Ochoa-Moreno, Misael Anaya-Montes, Luis Fernandes, Thaiza Gomes, Maria Do Carmo Leal, Cristóbal Cuadrado

Supplementary Methods 1. ReGHID survey

The *Redressing Gendered Health Inequalities of Displaced Women and Girls in contexts of Protracted Crisis in Central and South America* (ReGHID) is an interdisciplinary project, conducted between 2020 and 2023, by a consortium of academic partners in universities of Brazil, Colombia, El Salvador, Honduras and the United Kingdom. The overarching objective of ReGHID is to improve the delivery of sexual and reproductive health (SRH) care and rights of reproductive age women (aged 25-49) and adolescent girls (15-24) in situations of protracted displacement, to engender both evidence-based advocacy and concrete policy proposals for improving coverage of SRH services. The ReGHID survey and the analyses presented in this article contribute to one of the project's specific objectives, namely developing new quantitative empirical evidence on the impact of displacement on the SRH needs of women and adolescent girls from Venezuela living in Brazil.

Sample design:

We collected information from 2,012 Venezuelan migrant women between the ages of 15 and 49, living in the cities of Manaus and Boa Vista, who migrated to Brazil between 2018 and 2021. The sample was selected using a Respondent Driven Sampling (RDS) method. This method allows us to construct a representative sample, after adjusting for statistical weights, of the migrant Venezuelan women who arrived in Brazil between 2018 and 2021.[1] The Respondent Driven Sampling (RDS)[1] sampling method was used as a strategy to identify migrant women residing in the two Brazilian capitals Boa Vista and Manaus. RDS is an appropriate methodology for accessing difficult-to-reach populations, where the real size or location of the individuals to be accessed is not known. It is carried out through successive recruitment cycles and used mainly when study populations are difficult to access and the real size or location of the individuals to be accessed is not known. This method allows calculating the probability of selection based on the size of each participant's network.[2] Considering the connections between the participants, the sample can be considered as a cluster sample with different selection probabilities.[3]

Sample size. To determine our sample size of migrant Venezuelan women, we considered the use of health services in the last 15 days as a reference parameter. According to data from the National Health Survey from Brazil (PNS), the share of people using healthcare in the last 15 days was 18.6%.[4] The minimum sample size to estimate a proportion of 19% of healthcare users, with a bilateral error of 4% and a design effect of 2, corresponded to 730 women approximately.

Selection process. Before data collection began, we held meetings with partner institutions to identify women leaders from different social and geographical contexts. Then, the chain recruitment process started with the selection of women (called seeds) based on their place of residence, network size, time availability and having a mobile phone with internet access. However, as the interviews were conducted in person, having a mobile phone was not relevant for eligibility. In Manaus, 10 seeds were selected, of which only six were successful; and in

Manaus, 9 seeds were successful of 15 selected. The eligibility criteria were: (i) being a woman, (ii) Venezuelan, (iii) between 15 and 49 years old, (iv) having lived in Brazil for a maximum of 3 years, (v) presenting a valid invitation from the team to join the study, (vi) have not participated previously in the study.

After participating in the survey, each seed respondent received three invitations (coupons) to give to eligible women from the same population group. If any of them did not invite other participants, and/or those who were invited did not participate in the research, that branch of the invitation network was, therefore, interrupted. The process was repeated until the sample size in each of the two locations was reached. Each participant was assigned a unique code in order to identify all recruiter-participant pairs, to account for the effects of inviting participants with very similar characteristics[2]. Participants received financial aid to pay for transportation and food costs (primary incentive); moreover, recruiters received a secondary incentive for each guest that answered the questionnaire.

Data collection. The interviews took place between July and August 2021 in Manaus, and in September in Boa Vista, with the participation of 761 and 1,267 migrants respectively. After discarding invalid questionnaires and seeds, the resulting sample size was 2,012 women (1,257 from Boa Vista and 755 from Manaus).

All interviews were carried out in Spanish by Venezuelan interviewers trained by the project team. The interviews were carried out face-to-face. A structured questionnaire with closed questions, electronically programmed in the Redcap software, was used to collect data. The answers were marked by the interviewers in the electronic questionnaire using a tablet. In Boa Vista, the collections were carried out at the NGO *Mexendo a Panela* located in the Consolata Church and in Manaus at the NGO *Hermanitos*. These two organizations provide assistance and services to Venezuelan migrants in Brazil and receive a large flow of migrants daily. These organizations helped identify the seeds in each city.

As part of the interview, women were asked about their network, specifically: “how many Venezuelan women who migrated to Brazil in the last three years do you personally know?”; and, “how many of them are between 15 and 49 years of age?”. This information was used to estimate complex sampling weights and clusters.

Supplementary Methods 2. Variable construction

Variables	Questions used to construct health outcomes	
	ReGHID questionnaire	PNS questionnaire
Received care when sought	● During the last two weeks, did you attend a place, service or health provider seeking care related to your own health?<br>● Did you receive health care the first time that you sought it in the last two weeks?	● During the last two weeks, did you attend a place, service or health provider seeking care related to your own health?<br>● Did you receive health care the first time that you sought it in the last two weeks?
Did not pay for medications	● Do you have any of these chronic diseases confirmed by a doctor?<br>-Cardiovascular disease<br>-High blood pressure<br>-Lupus or scleroderma<br>-Hypothyroidism<br>-Diabetes or high sugar levels<br>-Renal disease<br>-Asthma<br>-High cholesterol<br>-Arthritis or rheumatism<br>-Depression<br>-Epilepsy<br>● Do you take medications regularly due to any of these chronic conditions?<br>● How do you obtain those medications here in Brazil?<br>**-National health system (SUS) network**<br>-You paid any amount for the medication<br>**-Donation**<br>**-NGO**	● Did a doctor or a health professional have diagnosed you with…?<br>-Hypertension<br>-Diabetes<br>-Asthma<br>-Depression<br>● Do you take any medications for …?<br>● Did you pay any amount for … medications?
Had a pap smear in the last three years	● When was the last time you had a preventive pap smear test?<br>**-In the last 12 months**<br>**-Between 1 and 2 years ago**<br>**-Between 2 and 3 years ago**<br>-Three or more years ago<br>-Never	● When was the last time you had a preventive pap smear test?<br>**-In the last 12 months**<br>**-Between 1 and 2 years ago**<br>**-Between 2 and 3 years ago**<br>-Three or more years ago<br>-Never
Antenatal care consultation	● Did you give birth in the last 12 months in Brazil?<br>● Did you attend a consultation to receive antenatal care during that pregnancy?	● On what date did you last give birth?<br>● When you were pregnant, did you attend any antenatal care consultation?
Timely pap smear results	● How long after undergoing your last pap smear test did you receive your results?<br>**-Less than a month after**<br>**-Between one and three months after**<br>-Between three and six months after<br>-More than six months after<br>-I have not received them yet<br>-Never received them<br>-Never went to collect them	● How long after undergoing your last pap smear test did you receive your results?<br>**-Less than a month after**<br>**-Between one and three months after**<br>-Between three and six months after<br>-More than six months after<br>-I have not received them yet<br>-Never received them<br>-Never went to collect them

Antenatal care as per guidelines	• During your prenatal care consultation(s), did you have a blood test, apart from a pregnancy test?<br>• During your prenatal care consultation(s), were you tested for Hepatitis B?<br>• During your prenatal care consultation(s), were you tested for HIV?	• During your prenatal care consultation(s), did you have a blood test, apart from a pregnancy test?<br>• During your prenatal care consultation(s), were you tested for Hepatitis B?<br>• During your prenatal care consultation(s), were you tested for HIV?
Has had STI	• Have you had any sexually transmitted infection in the last 30 days?	• Have been diagnosed with a sexually transmitted infection/disease in the last 12 months?
Good self-assessed health	• Considering health as a state of physical and mental well-being, and not only the absence of disease, what is the health status of ___?<br>**-Very good**<br>**-Good**<br>-Fair<br>-Bad<br>-Very bad	• Considering health as a state of physical and mental well-being, and not only the absence of disease, what is the health status of ___?<br>**-Very good**<br>**-Good**<br>-Fair<br>-Bad<br>-Very bad
Total income		

PNS: data from the Brazilian National Health Survey (*Pesquisa Nacional de Saúde*)
ReGHID: data from the ReGHID survey (Redressing Gendered Health Inequalities of Displaced Women and Girls in contexts of Protracted Crisis in Central and South America)

**Variables**	**Questions used to construct variables**	
	**ReGHID questionnaire**	**POF questionnaire**
Health expenditures	• Of the money spent last month, how much did your family spend out of pocket on medications, healthcare, dental care, hospital services, pharmacy, tests or others? Include all out of pocket payments made.	• Register the monetary expenditure on pharmaceutical products (for the purpose of preventing, treating or curing health problems) and healthcare products or services (related to health care), within the reference period.
Catastrophic health expenditures	• Total expenditure:<br>-Thinking about the last month that can be considered a typical or normal month, how much was spent for household consumption in total (services public, rent, food, house cleaning, shopping clothes, etc)?<br>• Health expenditure (see above)	• Total expenditure:<br>-The total of products acquired and the services used by the household for a period of reference.<br>They are organised according to the following groups: food, housing, clothing, transportation, hygiene and personal care, health care, education, recreation and culture, tobacco, personal services and other expenses.<br>• Health expenditure (see above)

POF: data from the Brazilian Household Budget Survey (*Pesquisa de Orçamentos Familiares*)
ReGHID: data from the ReGHID survey (Redressing Gendered Health Inequalities of Displaced Women and Girls in contexts of Protracted Crisis in Central and South America)

Supplementary Methods 3. VIF Analysis

High levels of multicollinearity, if present in our models, would primarily affect estimation efficiency by inflating our standard errors, thereby making it harder to find statistically significant effects of migration on health indicators where these exist. As some of our regressors may be correlated, we have checked for multicollinearity in the models by examining variance inflation factors (VIF).

The VIF is computed as:

$$VIF_j = \frac{1}{1 - R_j^2}$$

Where VIF_j is the variance inflation factor for the jth predictor and can take values from 1 to infinity; and R^2_j is the multiple correlation coefficient which gives the proportion of variance in the outcome associated with the p predictors. In general, a VIF=1 implies that the variables are not correlated; VIF between 1 and 5 indicates that the variables are moderately correlated; with VIF>5, the variables are highly correlated; and a VIF greater than 10 is evidence of multicollinearity.[5]

Overall, the analyses presented below strongly suggest that our main results are unaffected by multicollinearity. We present an example of the tests and their alternatives for different models. In our regression model for the variable CHE10, even though education categories show high VIF, the mean VIF indicates moderate correlation (VIF=4.39).

Variable	VIF	1/VIF
Migrant	1	0.999276
Manaus	1.06	0.947486
Age group		
20 - 29	2.44	0.410467
30 - 39	2.3	0.433977
40+	1.89	0.527722
Education		
Primary	11.87	0.084252
Secondary	19	0.052645
Higher	12.07	0.082837
Ethnicity		
Mixed	1.18	0.844524
Black	1.09	0.913439
Indigenous	1.1	0.912372
Other	1.03	0.975512
Household size	1.03	0.96622
Mean VIF	4.39	

We run the model without the education variables and the mean VIF is now close to 1 and the VIF for each of the covariates is below 5, implying moderate correlation. Importantly, the VIF for migrant is equal to 1, indicating no correlation with other variables.

Variable	VIF	1/VIF
Migrant	1	0.999391
Manaus	1.04	0.95825
Age group		
20 - 29	2.42	0.41366
30 - 39	2.26	0.442073
40+	1.88	0.533321
Ethnicity		
Mixed	1.18	0.846246
Black	1.09	0.91381
Indigenous	1.09	0.915396
Other	1.02	0.97579
Household size	1.03	0.968608
Mean VIF	1.4	

Finally, we compare the coefficients on being a migrant for both regressions and found no difference.

	Coefficient	S.E.	t	P>|t|	95% C.I.	
Excluding education	0.094	0.018	5.200	0.000	0.058	0.129
Including education	0.094	0.018	5.220	0.000	0.059	0.129

Supplementary Table 1. Descriptive statistics for migrants (ReGHID survey) and non-migrants (POF survey), before and after matching, for financial protection analyses

	Before matching				After matching			
	Migrants		Non-migrants		Migrants		Non-migrants	
Months since migration								
Up to 6	785/1997	(39.3)			784/1995	(39.3)		
Between 7 and 18	496/1997	(24.8)			495/1995	(24.8)		
Between 19 and 36	716/1997	(35.9)			716/1995	(35.9)		
Age	2012 29.5	(8.9)	1649 30.5	(9.8)	2010 29.5	(8.9)	1644 29.7	(9.3)
Age group								
< 20	268/2012	(13.3)	291/1649	(17.6)	267/2010	(13.3)	19/1644	(1.1)
20 - 29	845/2012	(42.0)	493/1649	(29.9)	844/2010	(42.0)	240/1644	(14.6)
30 - 39	564/2012	(28.0)	494/1649	(30.0)	564/2010	(28.1)	1130/1644	(68.8)
40+	335/2012	(16.7)	371/1649	(22.5)	335/2010	(16.7)	255/1644	(15.5)
Ethnicity								
White	577/2011	(28.7)	369/1644	(22.4)	577/2010	(28.7)	472/1644	(28.7)
Mixed	1286/2011	(63.9)	1140/1644	(69.3)	1286/2010	(64.0)	1052/1644	(64.0)
Black	71/2011	(3.5)	81/1644	(4.9)	71/2010	(3.5)	58/1644	(3.5)
Indigenous	62/2011	(3.1)	32/1644	(1.9)	61/2010	(3.0)	50/1644	(3.0)
Other	15/2011	(0.7)	22/1644	(1.3)	15/2010	(0.7)	12/1644	(0.7)
Education								
< primary	23/2011	(1.1)	64/1649	(3.9)	23/2010	(1.1)	19/1644	(1.1)
Primary	293/2011	(14.6)	517/1649	(31.4)	293/2010	(14.6)	240/1644	(14.6)
Secondary	1383/2011	(68.8)	673/1649	(40.8)	1382/2010	(68.8)	1130/1644	(68.8)
Higher	312/2011	(15.5)	395/1649	(24.0)	312/2010	(15.5)	255/1644	(15.5)
Household size	2012 2.6	(2.3)	1649 4.4	(1.9)	2010 2.6	(2.3)	1644 2.6	(2.3)
Boa Vista	1257/2012	(62.5)	591/1649	(35.8)	1255/2010	(62.4)	1027/1644	(62.4)
Manaus	755/2012	(37.5)	1058/1649	(64.2)	755/2010	(37.6)	617/1644	(37.6)
CHE 10%	278/1802	(15.4)	81/1644	(4.9)	277/1800	(15.4)	98/1639	(6.0)
CHE 25%	130/1802	(7.2)	10/1644	(0.6)	129/1800	(7.2)	9/1639	(0.6)
Health expenditure (R$)	1953 44.1	(154.3)	1644 116.6	(187.7)	1951 44.1	(154.4)	1639 90.7	(165.5)

POF: data from the Brazilian Household Budget Survey (*Pesquisa de Orçamentos Familiares*)
ReGHID: data from the ReGHID survey (Redressing Gendered Health Inequalities of Displaced Women and Girls in contexts of Protracted Crisis in Central and South America)
CHE: catastrophic health expenditures (healthcare expenditures exceed 10% or 25% of total household expenditure)
R$: Brazilian Real.

Supplementary Table 2. Descriptive statistics for migrants (ReGHID survey) and non-migrants (PNS survey), before and after matching, for healthcare access, care quality and health status analyses

	Before matching				After matching			
	Migrants		Non-migrants		Migrants		Non-migrants	
Months since migration								
Up to 6	785/1997	(39.3)			738/1868	(39.5)		
Between 7 and 18	496/1997	(24.8)			463/1868	(24.8)		
Between 19 and 36	716/1997	(35.9)			667/1868	(35.7)		
Age	2012 29.5	(8.9)	3047 31	(9.9)	1883 29.5	(8.8)	2233 29.4	(9.1)
Age group								
< 20	268/2012	(13.3)	494/3047	(16.2)	234/1883	(12.4)	277/2233	(12.4)
20 - 29	845/2012	(42.0)	925/3047	(30.4)	802/1883	(42.6)	951/2233	(42.6)
30 - 39	564/2012	(28.0)	879/3047	(28.8)	533/1883	(28.3)	632/2233	(28.3)
40+	335/2012	(16.7)	749/3047	(24.6)	314/1883	(16.7)	372/2233	(16.7)
Ethnicity								
White	577/2011	(28.7)	694/3047	(22.8)	538/1883	(28.6)	638/2233	(28.6)
Mixed	1286/2011	(63.9)	2068/3047	(67.9)	1203/1883	(63.9)	1427/2233	(63.9)
Black	71/2011	(3.5)	196/3047	(6.4)	70/1883	(3.7)	83/2233	(3.7)
Indigenous	62/2011	(3.1)	70/3047	(2.3)	57/1883	(3.0)	68/2233	(3.0)
Other	15/2011	(0.7)	19/3047	(0.6)	15/1883	(0.8)	18/2233	(0.8)
Education								
< Primary	23/2011	(1.1)	10/2240	(0.4)	22/1883	(1.2)	26/2233	(1.2)
Primary	293/2011	(14.6)	358/2240	(16.0)	268/1883	(14.2)	318/2233	(14.2)
Secondary	1383/2011	(68.8)	1229/2240	(54.9)	1293/1883	(68.7)	1533/2233	(68.7)
Higher	312/2011	(15.5)	643/2240	(28.7)	300/1883	(15.9)	356/2233	(15.9)
Lives with partner	1146/2011	(57.0)	1538/3047	(50.5)	1079/1883	(57.3)	1280/2233	(57.3)
Household size	2012 2.6	(2.3)	3040 4.3	(2.0)	1883 2.6	(2.3)	2233 2.6	(2.3)
Income (R$)								
No income	1106/1885	(58.7)	127/3040	(4.2)	1104/1883	(58.6)	1309/2233	(58.6)
< 500	611/1885	(32.4)	620/3040	(20.4)	611/1883	(32.4)	725/2233	(32.4)
500 - 1000	141/1885	(7.5)	1089/3040	(35.8)	141/1883	(7.5)	167/2233	(7.5)
> 1000	27/1885	(1.4)	1204/3040	(39.6)	27/1883	(1.4)	32/2233	(1.4)
Boa Vista	1257/2012	(62.5)	1442/3047	(47.3)	1188/1883	(63.1)	1409/2233	(63.1)
Manaus	755/2012	(37.5)	1605/3047	(52.7)	695/1883	(36.9)	824/2233	(36.9)
Received care when sought	638/670	(95.2)	392/564	(69.5)	604/634	(95.3)	260/419	(62.1)
Did not pay for medications	92/143	(64.3)	33/68	(48.5)	82/130	(63.1)	33/54	(61.5)
Self-assessed good health	1425/2009	(70.9)	2610/3047	(85.7)	1327/1880	(70.6)	1824/2233	(81.7)
Pap smear in last 3 years	465/947	(49.1)	725/847	(85.6)	432/891	(48.5)	619/724	(85.5)
Antenatal care consultation	281/311	(90.4)	85/93	(91.4)	260/289	(90.0)	72/85	(84.9)
Timely pap smear results	385/453	(85.0)	625/717	(87.2)	358/420	(85.2)	496/619	(80.1)
Antenatal care as per guidelines	236/267	(88.4)	71/93	(76.3)	217/246	(88.2)	71/85	(83.0)
Has STI	16/2012	(0.8)	12/1085	(1.1)	16/1883	(0.8)	1/863	(0.1)

PNS: data from the Brazilian National Health Survey (*Pesquisa Nacional de Saúde*)
ReGHID: data from the ReGHID survey (Redressing Gendered Health Inequalities of Displaced Women and Girls in contexts of Protracted Crisis in Central and South America)
R$: Brazilian Real.

Supplementary Table 3. Full estimation results: financial protection

	FINANCIAL PROTECTION					
	Catastrophic health expenditures 10%		Catastrophic health expenditures 25%		Health Expenditures	
	(Model 1)	(Model 2)	(Model 3)	(Model 4)	(Model 5)	(Model 6)
Migrant	0.095 (<0.0001)	0.006 (0.82)	0.066 (<0.0001)	0.051 (<0.0001)	-46.48 (<0.0001)	-71.08 (<0.0001)
	[0.06, 0.13]	[-0.04, 0.06]	[0.05, 0.08]	[0.03, 0.07]	[-65.20, -27.76]	[-96.59, -45.57]
Migrant*Manaus		0.246 (<0.0001)		0.041 (0.009)		66.52 (0.0001)
		[0.18, 0.31]		[0.01, 0.07]		[33.49, 99.56]
Manaus	0.067 (0.0003)	-0.047 (0.0907)	0.019 (0.009)	-0.000 (0.96)	31.21 (0.003)	-1.14 (0.95)
	[0.03, 0.10]	[-0.10, 0.01]	[0.00, 0.03]	[-0.01, 0.01]	[10.76, 51.67]	[-33.71, 31.44]
Age						
20-29	0.011 (0.75)	0.007 (0.8290)	0.012 (0.17)	0.011 (0.18)	11.19 (0.18)	10.28 (0.22)
	[-0.06, 0.08]	[-0.06, 0.07]	[-0.00, 0.03]	[-0.01, 0.03]	[-5.35, 27.74]	[-6.13, 26.68]
30-39	0.058 (0.14)	0.057 (0.1398)	0.020 (0.094)	0.020 (0.094)	28.67 (0.033)	28.40 (0.033)
	[-0.02, 0.13]	[-0.02, 0.13]	[-0.00, 0.04]	[-0.00, 0.04]	[2.35, 54.99]	[2.16, 54.65]
40+	0.008 (0.81)	0.005 (0.8723)	0.009 (0.39)	0.008 (0.42)	11.54 (0.22)	10.42 (0.28)
	[-0.06, 0.07]	[-0.06, 0.07]	[-0.01, 0.03]	[-0.01, 0.03]	[-7.00, 30.08]	[-8.35, 29.19]
Education (base = no ed)						
Primary	-0.032 (0.52)	-0.046 (0.3453)	-0.008 (0.81)	-0.010 (0.75)	33.34 (0.029)	29.77 (0.045)
	[-0.13, 0.06]	[-0.14, 0.05]	[-0.07, 0.05]	[-0.07, 0.05]	[3.47, 63.22]	[0.57, 58.97]
Secondary	-0.021 (0.65)	-0.040 (0.3998)	-0.019 (0.54)	-0.022 (0.48)	42.33 (0.002)	37.30 (0.004)
	[-0.11, 0.07]	[-0.13, 0.05]	[-0.08, 0.04]	[-0.08, 0.04]	[16.07, 68.60]	[12.10, 62.51]
Higher	-0.009 (0.86)	-0.037 (0.4682)	-0.025 (0.42)	-0.030 (0.34)	97.88 (<0.0001)	89.97 (0.0001)
	[-0.11, 0.09]	[-0.14, 0.06]	[-0.09, 0.04]	[-0.09, 0.03]	[50.94, 144.82]	[45.29, 134.66]
Ethnicity (base=White)						
Mixed	0.014 (0.38)	0.011 (0.4839)	0.000 (0.96)	-0.000 (0.97)	-11.37 (0.27)	-12.26 (0.23)
	[-0.02, 0.05]	[-0.02, 0.04]	[-0.01, 0.01]	[-0.01, 0.01]	[-31.55, 8.76]	[-32.37, 7.86]
Black	0.038 (0.44)	0.042 (0.3849)	0.059 (0.22)	0.060 (0.22)	-18.88 (0.20)	-17.82 (0.22)
	[-0.06, 0.14]	[-0.05, 0.14]	[-0.04, 0.16]	[-0.04, 0.16]	[-47.69, 9.94]	[-46.52, 10.88]
Indigenous	-0.036 (0.13)	-0.043 (0.1127)	0.003 (0.86)	0.002 (0.92)	-36.26 (0.007)	-38.55 (0.005)
	[-0.08, 0.01]	[-0.10, 0.01]	[-0.03, 0.04]	[-0.03, 0.04]	[-62.72, -9.80]	[-65.44, -11.66]
Other	-0.014 (0.81)	0.014 (0.7833)	0.034 (0.48)	0.039 (0.41)	-14.68 (0.66)	-7.35 (0.82)
	[-0.13, 0.10]	[-0.09, 0.11]	[-0.06, 0.13]	[-0.05, 0.13]	[-79.77, 50.41]	[-69.29, 54.60]
Household size	-0.003 (0.17)	-0.003 (0.17)	0.001 (0.63)	0.001 (0.64)	13.26 (0.0001)	13.25 (<0.0001)
	[-0.01, 0.00]	[-0.01, 0.00]	[-0.00, 0.00]	[-0.00, 0.00]	[6.60, 19.92]	[7.00, 19.51]
Constant	0.032 (0.58)	0.098 (0.10)	0.001 (0.99)	0.012 (0.70)	-10.32 (0.55)	8.22 (0.64)
	[-0.08, 0.15]	[-0.02, 0.22]	[-0.06, 0.06]	[-0.05, 0.07]	[-44.33, 23.69]	[-26.05, 42.49]
Observations	3,439	3,439	3,439	3,439	3,590	3,590
R-squared	0.045	0.082	0.038	0.040	0.086	0.096
F-stat	8.330	12.38	9.523	9.074	8.691	11.22
Prob > F	<0.0001	<0.0001	<0.0001	<0.0001	<0.0001	<0.0001

Robust p values in parentheses

95% CI in brackets

R$: Brazilian Real.

Migrant*Manaus is the interaction term of migrant and Manaus. Refers to migrants living in the city of Manaus

Models 1 to 4 present the probabilities of incurring catastrophic health expenditures (10% or 25%) associated with being a migrant (vs local), being a migrant in Manaus (vs migrant in Boa Vista) or living in Manaus (vs living in Boa Vista). Models 5-6 show the difference in healthcare expenditures in R$ associated with being a migrant in Manaus, and living in Manaus (migrant and non-migrant). All models are adjusted for sociodemographic characteristics of women.

F-stat: Tests whether there is no predictive relationship between the regressors and the outcome in the models. We reject the null hypothesis of no predictive relationship with values of Prob > F smaller than 0.05.

Supplementary Table 4. Full estimation results: healthcare access, quality and health status (general health outcomes)

	ACCESS TO CARE, QUALITY OF CARE AND HEALTH STATUS					
	General health					
	Sought and received care		Publicly provided meds		Good self-assessed health	
	(Model 1)	(Model 2)	(Model 3)	(Model 4)	(Model 5)	(Model 6)
Migrant	0.270 (0.0008)	0.296 (0.008)	0.124 (0.23)	0.060 (0.66)	-0.111 (0.002)	-0.066 (0.23)
	[0.11, 0.43]	[0.08, 0.51]	[-0.08, 0.33]	[-0.21, 0.33]	[-0.18, -0.04]	[-0.17, 0.04]
Migrant*Manaus		-0.075 (0.65)		0.147 (0.37)		-0.122 (0.15)
		[-0.40, 0.25]		[-0.18, 0.47]		[-0.29, 0.04]
Manaus	-0.051 (0.47)	-0.011 (0.94)	-0.530 (<0.0001)	-0.628 (<0.0001)	-0.072 (0.068)	-0.016 (0.83)
	[-0.19, 0.09]	[-0.31, 0.29]	[-0.69, -0.37]	[-0.89, -0.36]	[-0.15, 0.01]	[-0.15, 0.12]
Age						
20-29	-0.191 (0.049)	-0.180 (0.064)	-0.206 (0.17)	-0.195 (0.21)	-0.030 (0.67)	-0.021 (0.76)
	[-0.38, -0.00]	[-0.37, 0.01]	[-0.50, 0.09]	[-0.50, 0.11]	[-0.17, 0.11]	[-0.16, 0.11]
30-39	-0.168 (0.18)	-0.167 (0.19)	-0.171 (0.26)	-0.175 (0.27)	-0.103 (0.18)	-0.101 (0.18)
	[-0.41, 0.08]	[-0.42, 0.08]	[-0.47, 0.13]	[-0.49, 0.14]	[-0.25, 0.05]	[-0.25, 0.05]
40+	-0.127 (0.18)	-0.127 (0.18)	-0.012 (0.93)	-0.022 (0.89)	-0.150 (0.064)	-0.145 (0.069)
	[-0.31, 0.06]	[-0.31, 0.06]	[-0.30, 0.28]	[-0.33, 0.28]	[-0.31, 0.01]	[-0.30, 0.01]
Ethnicity (white base)						
Mixed	0.287 (0.009)	0.280 (0.006)	0.043 (0.64)	0.047 (0.61)	-0.034 (0.35)	-0.035 (0.36)
	[0.07, 0.50]	[0.08, 0.48]	[-0.14, 0.22]	[-0.13, 0.22]	[-0.11, 0.04]	[-0.11, 0.04]
Black	0.161 (0.28)	0.151 (0.28)	-0.028 (0.86)	-0.025 (0.87)	-0.050 (0.43)	-0.060 (0.34)
	[-0.13, 0.45]	[-0.12, 0.42]	[-0.34, 0.29]	[-0.34, 0.29]	[-0.18, 0.08]	[-0.18, 0.06]
Indigenous	0.211 (0.032)	0.207 (0.026)	0.015 (0.95)	-0.006 (0.98)	-0.184 (0.21)	-0.177 (0.22)
	[0.02, 0.40]	[0.03, 0.39]	[-0.42, 0.45]	[-0.45, 0.44]	[-0.47, 0.11]	[-0.46, 0.11]
Other	0.213 (0.029)	0.212 (0.026)	0.758 (0.001)	0.804 (0.001)	-0.291 (0.095)	-0.309 (0.081)
	[0.02, 0.40]	[0.03, 0.40]	[0.30, 1.22]	[0.33, 1.28]	[-0.63, 0.05]	[-0.66, 0.04]
Schooling (base = no ed)						
Primary	0.022 (0.67)	0.020 (0.70)	-0.112 (0.33)	-0.136 (0.23)	0.297 (0.13)	0.317 (0.11)
	[-0.08, 0.12]	[-0.08, 0.12]	[-0.34, 0.11]	[-0.36, 0.09]	[-0.09, 0.68]	[-0.07, 0.71]
Secondary	-0.056 (0.19)	-0.055 (0.21)	-0.106 (0.37)	-0.135 (0.25)	0.340 (0.068)	0.353 (0.06)
	[-0.14, 0.03]	[-0.14, 0.03]	[-0.34, 0.13]	[-0.37, 0.10]	[-0.03, 0.71]	[-0.02, 0.73]
Higher	0.058 (0.47)	0.067 (0.47)	0.037 (0.76)	-0.025 (0.85)	0.392 (0.037)	0.415 (0.030)
	[-0.10, 0.21]	[-0.09, 0.23]	[-0.21, 0.28]	[-0.28, 0.23]	[0.02, 0.76]	[0.04, 0.79]
Married	-0.183 (0.007)	-0.178 (0.013)	0.044 (0.60)	0.039 (0.64)	-0.023 (0.54)	-0.019 (0.61)
	[-0.32, -0.05]	[-0.32, -0.04]	[-0.12, 0.21]	[-0.13, 0.20]	[-0.10, 0.05]	[-0.09, 0.05]
HH monthly income (base=0)						
< 500	0.174 (0.015)	0.185 (0.014)	-0.031 (0.72)	-0.047 (0.60)	0.005 (0.90)	0.024 (0.56)
	[0.03, 0.31]	[0.04, 0.33]	[-0.21, 0.14]	[-0.22, 0.13]	[-0.07, 0.08]	[-0.06, 0.10]
500 - 1000	0.206 (0.011)	0.222 (0.009)	0.034 (0.81)	0.031 (0.83)	-0.011 (0.80)	0.014 (0.77)
	[0.05, 0.36]	[0.05, 0.39]	[-0.24, 0.31]	[-0.25, 0.31]	[-0.10, 0.07]	[-0.08, 0.11]
> 1000	0.323 (0.005)	0.343 (0.005)	0.187 (0.64)	0.149 (0.70)	0.056 (0.43)	0.094 (0.22)
	[0.10, 0.55]	[0.10, 0.58]	[-0.59, 0.97]	[-0.63, 0.92]	[-0.08, 0.20]	[-0.06, 0.24]
Household size	0.009 (0.14)	0.009 (0.14)	0.000 (0.99)	0.003 (0.90)	-0.002 (0.71)	-0.002 (0.64)
	[-0.00, 0.02]	[-0.00, 0.02]	[-0.04, 0.04]	[-0.04, 0.05]	[-0.01, 0.01]	[-0.01, 0.01]
Constant	0.692 (<0.0001)	0.668 (0.0005)	0.862 (<0.0001)	0.943 (<0.0001)	0.620 (0.002)	0.570 (0.007)
	[0.39, 1.00]	[0.29, 1.05]	[0.55, 1.17]	[0.59, 1.30]	[0.23, 1.01]	[0.15, 0.99]
Observations	1,053	1,053	184	184	4,113	4,113
R-squared	0.453	0.454	0.438	0.441	0.059	0.063
F-stat	1.313	1.582	16.10	21.79	3.953	5.441
Prob > F	0.18	0.06	<0.0001	<0.0001	<0.0001	<0.0001

Robust p values in parentheses; 95% CI in brackets
Migrant*Manaus is the interaction term of migrant and Manaus. Refers to migrants living in the city of Manaus
This table presents the probabilities of receiving each health service associated with being a migrant (vs local), being a migrant in Manaus (vs migrant in Boa Vista) or living in Manaus (vs living in Boa Vista).
All models are adjusted for sociodemographic characteristics of women.
F-stat: Tests whether there is no predictive relationship between the regressors and the outcome in the models. We reject the null hypothesis of no predictive relationship with values of Prob > F smaller than 0.05.

Supplementary Table 5. Full estimation results: healthcare access and quality (sexual and reproductive health outcomes)

	HEALTHCARE ACCESS AND QUALITY							
	Sexual and reproductive health							
	Pap smear in last 3 years		Antenatal care consultation		Timely pap smear results		Antenatal care as per guidelines	
	(Model 7)	(Model 8)	(Model 9)	(Model 10)	(Model 11)	(Model 12)	(Model 13)	(Model 14)
Migrant	-0.368 (<0.0001)	-0.437 (<0.0001)	0.141 (0.26)	0.358 (0.065)	0.068 (0.39)	0.056 (0.56)	0.148 (0.24)	0.384 (0.046)
	[-0.45, -0.29]	[-0.55, -0.33]	[-0.11, 0.39]	[-0.02, 0.74]	[-0.09, 0.22]	[-0.14, 0.25]	[-0.10, 0.40]	[0.01, 0.76]
Migrant*Manaus		0.162 (0.068)		-0.507 (0.027)		0.024 (0.84)		-0.553 (0.016)
		[-0.01, 0.34]		[-0.96, -0.06]		[-0.22, 0.27]		[-1.00, -0.11]
Manaus	0.104 (0.017)	0.022 (0.77)	0.153 (0.029)	0.449 (0.014)	-0.009 (0.90)	-0.020 (0.87)	0.181 (0.022)	0.493 (0.007)
	[0.02, 0.19]	[-0.13, 0.17]	[0.02, 0.29]	[0.09, 0.81]	[-0.16, 0.14]	[-0.25, 0.21]	[0.03, 0.33]	[0.13, 0.85]
Age								
20-29			-0.123 (0.11)	-0.043 (0.29)			-0.133 (0.11)	-0.043 (0.36)
			[-0.27, 0.03]	[-0.12, 0.04]			[-0.30, 0.03]	[-0.14, 0.05]
30-39	0.058 (0.33)	0.060 (0.31)	-0.076 (0.28)	0.006 (0.93)	-0.084 (0.26)	-0.083 (0.26)	-0.125 (0.14)	-0.032 (0.71)
	[-0.06, 0.17]	[-0.06, 0.17]	[-0.21, 0.06]	[-0.13, 0.14]	[-0.23, 0.06]	[-0.23, 0.06]	[-0.29, 0.04]	[-0.20, 0.14]
40+	0.187 (0.003)	0.186 (0.003)	-0.306 (0.044)	-0.226 (0.16)	0.005 (0.93)	0.006 (0.93)	-0.279 (0.10)	-0.163 (0.32)
	[0.06, 0.31]	[0.06, 0.31]	[-0.60, -0.01]	[-0.54, 0.09]	[-0.12, 0.13]	[-0.12, 0.13]	[-0.61, 0.05]	[-0.49, 0.16]
Ethnicity (white base)								
Mixed	0.014 (0.67)	0.015 (0.62)	-0.088 (0.18)	-0.064 (0.19)	0.077 (0.39)	0.077 (0.38)	-0.113 (0.12)	-0.079 (0.14)
	[-0.05, 0.08]	[-0.04, 0.07]	[-0.22, 0.04]	[-0.16, 0.03]	[-0.10, 0.25]	[-0.10, 0.25]	[-0.26, 0.03]	[-0.19, 0.03]
Black	-0.055 (0.53)	-0.048 (0.60)	-0.015 (0.88)	-0.013 (0.89)	-0.064 (0.65)	-0.062 (0.66)	-0.127 (0.28)	-0.119 (0.31)
	[-0.23, 0.12]	[-0.23, 0.13]	[-0.21, 0.18]	[-0.20, 0.18]	[-0.34, 0.21]	[-0.34, 0.21]	[-0.36, 0.10]	[-0.35, 0.11]
Indigenous	-0.100 (0.40)	-0.110 (0.37)	0.067 (0.30)	0.070 (0.32)	0.076 (0.63)	0.075 (0.64)	-0.178 (0.21)	-0.167 (0.21)
	[-0.33, 0.13]	[-0.35, 0.13]	[-0.06, 0.19]	[-0.07, 0.21]	[-0.24, 0.39]	[-0.24, 0.39]	[-0.46, 0.10]	[-0.43, 0.10]
Other	-0.236 (0.41)	-0.207 (0.44)	-0.014 (0.93)	0.034 (0.72)	0.018 (0.93)	0.021 (0.92)	-0.021 (0.91)	0.038 (0.73)
	[-0.79, 0.32]	[-0.74, 0.32]	[-0.33, 0.30]	[-0.15, 0.22]	[-0.41, 0.45]	[-0.41, 0.45]	[-0.38, 0.34]	[-0.18, 0.25]
Schooling (base = no ed)								
Primary	-0.035 (0.70)	-0.073 (0.40)	-0.053 (0.72)	-0.054 (0.72)	-0.286 (0.09)	-0.291 (0.07)	-0.181 (0.22)	-0.236 (0.08)
	[-0.21, 0.14]	[-0.25, 0.10]	[-0.34, 0.23]	[-0.35, 0.24]	[-0.61, 0.04]	[-0.60, 0.02]	[-0.47, 0.11]	[-0.50, 0.03]
Secondary	-0.021 (0.78)	-0.041 (0.60)	-0.061 (0.69)	-0.037 (0.82)	-0.168 (0.08)	-0.170 (0.08)	-0.264 (0.07)	-0.276 (0.045)
	[-0.17, 0.13]	[-0.20, 0.11]	[-0.36, 0.24]	[-0.35, 0.28]	[-0.36, 0.02]	[-0.36, 0.02]	[-0.55, 0.02]	[-0.54, -0.01]
Higher	0.027 (0.71)	-0.013 (0.87)	-0.028 (0.88)	0.024 (0.90)	-0.105 (0.15)	-0.111 (0.16)	-0.160 (0.34)	-0.135 (0.41)
	[-0.11, 0.16]	[-0.16, 0.14]	[-0.39, 0.33]	[-0.36, 0.41]	[-0.25, 0.04]	[-0.26, 0.04]	[-0.49, 0.17]	[-0.46, 0.18]

Continues on next page

	HEALTHCARE ACCESS AND QUALITY							
	Sexual and reproductive health							
	Pap smear in last 3 years		Antenatal care consultation		Timely pap smear results		Antenatal care as per guidelines	
	(Model 7)	(Model 8)	(Model 9)	(Model 10)	(Model 11)	(Model 12)	(Model 13)	(Model 14)
Married	0.101 (0.019)	0.100 (0.020)	-0.137 (0.09)	-0.090 (0.010)	-0.093 (0.28)	-0.093 (0.28)	-0.092 (0.30)	-0.042 (0.5146)
	[0.02, 0.19]	[0.02, 0.18]	[-0.30, 0.02]	[-0.20, 0.02]	[-0.26, 0.07]	[-0.26, 0.07]	[-0.27, 0.08]	[-0.17, 0.08]
HH monthly income (base=0)								
< 500	0.024 (0.60)	-0.008 (0.87)	0.165 (0.012)	0.261 (0.004)	-0.065 (0.38)	-0.069 (0.34)	0.130 (0.09)	0.233 (0.019)
	[-0.07, 0.11]	[-0.10, 0.08]	[0.04, 0.29]	[0.08, 0.44]	[-0.21, 0.08]	[-0.21, 0.07]	[-0.02, 0.28]	[0.04, 0.43]
500 - 1000	0.011 (0.84)	-0.026 (0.63)	0.077 (0.22)	0.212 (0.022)	0.037 (0.65)	0.032 (0.68)	0.066 (0.31)	0.209 (0.031)
	[-0.10, 0.12]	[-0.13, 0.08]	[-0.05, 0.20]	[0.03, 0.39]	[-0.12, 0.20]	[-0.12, 0.19]	[-0.06, 0.19]	[0.02, 0.40]
> 1000	-0.036 (0.75)	-0.083 (0.46)	0.115 (0.030)	0.257 (0.003)	-0.088 (0.51)	-0.095 (0.46)	-0.192 (0.39)	-0.036 (0.88)
	[-0.26, 0.19]	[-0.30, 0.14]	[0.01, 0.22]	[0.09, 0.43]	[-0.35, 0.18]	[-0.35, 0.16]	[-0.63, 0.25]	[-0.52, 0.45]
Household size	-0.020 (0.13)	-0.020 (0.15)	0.002 (0.78)	0.003 (0.72)	0.025 (0.008)	0.025 (0.0076)	-0.003 (0.81)	-0.000 (0.99)
	[-0.05, 0.01]	[-0.05, 0.01]	[-0.01, 0.02]	[-0.01, 0.02]	[0.01, 0.04]	[0.01, 0.04]	[-0.03, 0.02]	[-0.02, 0.02]
Constant	0.718 (<0.0001)	0.792 (<0.0001)	0.947 (<0.0001)	0.626 (0.010)	0.963 (<0.0001)	0.972 (<0.0001)	1.141 (<0.0001)	0.826 (0.0003)
	[0.55, 0.88]	[0.60, 0.98]	[0.60, 1.29]	[0.15, 1.10]	[0.81, 1.12]	[0.77, 1.17]	[0.82, 1.47]	[0.38, 1.27]
Observations	1,615	1,615	374	374	1,039	1,039	331	331
R-squared	0.211	0.216	0.224	0.301	0.078	0.078	0.209	0.290
F-stat	24.63	25.86	1.512	1.889	3.285	3.071	2.265	2.446
Prob > F	<0.0001	<0.0001	0.09	0.018	<0.0001	<0.0001	0.004	0.001

Robust p values in parentheses
95% CI in brackets
Migrant*Manaus is the interaction term of migrant and Manaus. Refers to migrants living in the city of Manaus
This table presents the probabilities of receiving each health service associated with being a migrant (vs local), being a migrant in Manaus (vs migrant in Boa Vista) or living in Manaus (vs living in Boa Vista).
All models are adjusted for sociodemographic characteristics of women.
F-stat: Tests whether there is no predictive relationship between the regressors and the outcome in the models. We reject the null hypothesis of no predictive relationship with values of Prob > F smaller than 0.05.

Supplementary Table 6. Full estimation results: financial protection according to time since migration

	FINANCIAL PROTECTION		
	**Catastrophic health expenditure 10%**	**Catastrophic health expenditure 25%**	**Health Expenditure (R$)**
	**(Model 1)**	**(Model 2)**	**(Model 3)**
Duration (base=0)			
<= 6 months	0.020 (0.3659)	0.046 (<0.0001)	-66.02 (<0.0001)
	[-0.02, 0.06]	[0.03, 0.06]	[-86.48, -45.55]
7-18 months	0.118 (<0.0001)	0.082 (<0.0001)	-50.80 (<0.0001)
	[0.07, 0.16]	[0.05, 0.11]	[-71.22, -30.38]
>18 months	0.159 (<0.0001)	0.078 (<0.0001)	-22.13 (0.05)
	[0.12, 0.20]	[0.06, 0.10]	[-44.45, 0.20]
Manaus	0.041 (0.033)	0.013 (0.07)	23.82 (0.027)
	[0.00, 0.08]	[-0.00, 0.03]	[2.70, 44.95]
Age			
20-29	0.007 (0.84)	0.011 (0.19)	10.35 (0.22)
	[-0.06, 0.07]	[-0.01, 0.03]	[-6.10, 26.80]
30-39	0.051 (0.19)	0.018 (0.12)	26.81 (0.046)
	[-0.03, 0.13]	[-0.00, 0.04]	[0.52, 53.09]
40+	-0.001 (0.97)	0.007 (0.49)	9.08 (0.34)
	[-0.07, 0.06]	[-0.01, 0.03]	[-9.58, 27.74]
Schooling (base = no ed)			
Primary	-0.038 (0.44)	-0.010 (0.76)	31.72 (0.043)
	[-0.14, 0.06]	[-0.07, 0.05]	[1.07, 62.37]
Secondary	-0.027 (0.57)	-0.021 (0.51)	41.39 (0.003)
	[-0.12, 0.07]	[-0.08, 0.04]	[14.41, 68.36]
Higher	-0.017 (0.74)	-0.028 (0.38)	96.10 (0.0001)
	[-0.12, 0.09]	[-0.09, 0.03]	[48.76, 143.44]
Ethnicity (white base)			
Mixed	0.016 (0.33)	0.001 (0.91)	-10.92 (0.29)
	[-0.02, 0.05]	[-0.01, 0.01]	[-31.09, 9.25]
Black	0.044 (0.38)	0.060 (0.22)	-16.63 (0.25)
	[-0.05, 0.14]	[-0.04, 0.16]	[-45.22, 11.96]
Indigenous	-0.049 (0.042)	0.000 (0.10)	-40.10 (0.003)
	[-0.10, -0.00]	[-0.03, 0.04]	[-66.74, -13.45]
Other	0.003 (0.95)	0.039 (0.41)	-12.66 (0.69)
	[-0.11, 0.11]	[-0.05, 0.13]	[-75.77, 50.45]
Household size	-0.003 (0.19)	0.001 (0.59)	13.46 (0.0001)
	[-0.01, 0.00]	[-0.00, 0.00]	[6.95, 19.97]
Constant	0.051 (0.38)	0.005 (0.86)	-5.90 (0.73)
	[-0.06, 0.17]	[-0.06, 0.07]	[-39.92, 28.13]
Observations	3,427	3,427	3,578
R-squared	0.062	0.041	0.092
F-stat	9.674	8.316	9.658
Prob > F	<0.0001	<0.0001	<0.0001
F-stat d1=d2 d1=d3	26.92	3.99	11.34
p>F d1=d2 d1=d3	<0.0001	0.019	<0.0001
F-stat d2=d3	2.673	0.0323	8.686
p>F d2=d3	0.10	0.86	0.003

Robust p values in parentheses; 95% CI in brackets

Models 1 and 2 present the probabilities of incurring catastrophic health expenditures (10% or 25%) associated with the time since arriving to Brazil: less than 6 months between 7 and 18 months, and between 19 and 36 months. Model 3 shows the difference in healthcare expenditures in R$ associated with time since arriving to Brazil.

All models are adjusted for sociodemographic characteristics of women.

F-stat: Tests whether there is no predictive relationship between the regressors and the outcome in our model. We reject the null hypothesis of no predictive relationship with values of Prob > F under 0.05.

p>F d1=d2 d1=d3 tests whether being in Brazil for 6 months or less (d1) is statistically different than being for more than 6 months (d2 & d3) for each outcome.

p>F d2=d3 tests whether being in Brazil for 7-18 months (d2) is statistically different than being for 19-36 months for each outcome.

Supplementary Table 7. Full estimation results: healthcare access, quality and health status according to time since migration

	ACCESS TO CARE, QUALITY OF CARE AND HEALTH STATUS						
	General health			Sexual and reproductive health			
	Received care when sought	Did not pay for medications	Self-assessed good health	Pap smear in last 3 years	Antenatal care consultation	Timely pap smear results	Antenatal care as per guidelines
	(Model 1)	(Model 2)	(Model 3)	(Model 4)	(Model 5)	(Model 6)	(Model 7)
Time since migrating							
<= 6 months	0.278 (0.002)	0.090 (0.44)	-0.113 (0.010)	-0.379 (<0.0001)	0.112 (0.50)	0.097 (0.27)	0.162 (0.34)
	[0.10, 0.46]	[-0.14, 0.32]	[-0.20, -0.03]	[-0.48, -0.28]	[-0.21, 0.44]	[-0.07, 0.27]	[-0.17, 0.50]
7-18 months	0.268 (0.0008)	0.147 (0.29)	-0.097 (0.015)	-0.320 (<0.0001)	0.189 (0.16)	0.078 (0.36)	0.147 (0.29)
	[0.11, 0.43]	[-0.13, 0.42]	[-0.18, -0.02]	[-0.42, -0.22]	[-0.07, 0.45]	[-0.09, 0.25]	[-0.13, 0.42]
18-36 months	0.255 (0.0008)	0.148 (0.21)	-0.120 (0.001)	-0.386 (<0.0001)	0.132 (0.20)	0.040 (0.62)	0.142 (0.17)
	[0.11, 0.40]	[-0.08, 0.38]	[-0.19, -0.05]	[-0.47, -0.30]	[-0.07, 0.33]	[-0.12, 0.20]	[-0.06, 0.35]
Manaus	-0.048 (0.52)	-0.524 (<0.0001)	-0.071 (0.08)	0.101 (0.023)	0.151 (0.05)	-0.002 (0.98)	0.183 (0.032)
	[-0.19, 0.10]	[-0.70, -0.35]	[-0.15, 0.01]	[0.01, 0.19]	[-0.00, 0.30]	[-0.15, 0.15]	[0.02, 0.35]
Age							
20-29	-0.190 (0.05)	-0.194 (0.22)	-0.028 (0.69)		-0.120 (0.11)		-0.132 (0.11)
	[-0.38, 0.00]	[-0.51, 0.12]	[-0.17, 0.11]		[-0.27, 0.03]		[-0.29, 0.03]
30-39	-0.168 (0.18)	-0.158 (0.33)	-0.102 (0.19)	0.059 (0.32)	-0.073 (0.28)	-0.079 (0.29)	-0.124 (0.14)
	[-0.41, 0.08]	[-0.48, 0.16]	[-0.25, 0.05]	[-0.06, 0.18]	[-0.20, 0.06]	[-0.23, 0.07]	[-0.29, 0.04]
40+	-0.132 (0.17)	0.015 (0.92)	-0.147 (0.073)	0.187 (0.004)	-0.307 (0.045)	0.013 (0.84)	-0.280 (0.10)
	[-0.32, 0.05]	[-0.30, 0.33]	[-0.31, 0.01]	[0.06, 0.31]	[-0.61, -0.01]	[-0.11, 0.14]	[-0.62, 0.06]
Ethnicity (white base)							
Mixed	0.288 (0.008)	0.043 (0.64)	-0.036 (0.34)	0.015 (0.63)	-0.086 (0.18)	0.075 (0.41)	-0.112 (0.12)
	[0.08, 0.50]	[-0.14, 0.23]	[-0.11, 0.04]	[-0.05, 0.08]	[-0.21, 0.04]	[-0.10, 0.25]	[-0.25, 0.03]
Black	0.161 (0.28)	-0.005 (0.98)	-0.052 (0.42)	-0.057 (0.52)	-0.005 (0.96)	-0.069 (0.63)	-0.129 (0.28)
	[-0.13, 0.45]	[-0.34, 0.33]	[-0.18, 0.07]	[-0.23, 0.12]	[-0.20, 0.19]	[-0.35, 0.21]	[-0.36, 0.10]
Indigenous	0.214 (0.031)	0.015 (0.95)	-0.183 (0.22)	-0.100 (0.40)	0.066 (0.33)	0.076 (0.64)	-0.179 (0.21)
	[0.02, 0.41]	[-0.43, 0.46]	[-0.47, 0.11]	[-0.33, 0.13]	[-0.07, 0.20]	[-0.24, 0.39]	[-0.46, 0.10]
Other	0.215 (0.023)	0.784 (0.001)	-0.291 (0.10)	-0.232 (0.41)	0.005 (0.98)	0.004 (0.99)	-0.025 (0.89)
	[0.03, 0.40]	[0.31, 1.26]	[-0.63, 0.05]	[-0.79, 0.33]	[-0.33, 0.34]	[-0.42, 0.43]	[-0.39, 0.34]
Schooling (base = no ed)							
Primary	0.021 (0.68)	-0.100 (0.47)	0.313 (0.11)	-0.033 (0.71)	-0.040 (0.79)	-0.282 (0.09)	-0.191 (0.20)
	[-0.08, 0.12]	[-0.37, 0.17]	[-0.07, 0.70]	[-0.21, 0.14]	[-0.33, 0.25]	[-0.61, 0.05]	[-0.48, 0.10]

Continues on next page

Secondary	-0.059 (0.17)	-0.073 (0.58)	0.355 (0.06)	-0.019 (0.81)	-0.054 (0.74)	-0.164 (0.09)	-0.275 (0.077)
	[-0.14, 0.03]	[-0.34, 0.19]	[-0.01, 0.72]	[-0.17, 0.13]	[-0.37, 0.26]	[-0.35, 0.03]	[-0.58, 0.03]
Higher	0.058 (0.47)	0.062 (0.66)	0.407 (0.030)	0.027 (0.71)	-0.026 (0.89)	-0.101 (0.17)	-0.171 (0.32)
	[-0.10, 0.21]	[-0.22, 0.34]	[0.04, 0.78]	[-0.11, 0.17]	[-0.39, 0.34]	[-0.25, 0.04]	[-0.51, 0.16]
Married	-0.184 (0.007)	0.047 (0.58)	-0.023 (0.54)	0.099 (0.022)	-0.136 (0.11)	-0.092 (0.28)	-0.093 (0.32)
	[-0.32, -0.05]	[-0.12, 0.21]	[-0.10, 0.05]	[0.01, 0.18]	[-0.30, 0.03]	[-0.26, 0.08]	[-0.28, 0.09]
HH monthly income (base=0, R$)							
< 500	0.180 (0.015)	-0.052 (0.57)	0.004 (0.92)	0.023 (0.63)	0.163 (0.037)	-0.055 (0.47)	0.134 (0.13)
	[0.03, 0.32]	[-0.23, 0.13]	[-0.07, 0.08]	[-0.07, 0.12]	[0.01, 0.32]	[-0.20, 0.09]	[-0.04, 0.31]
500 - 1000	0.211 (0.010)	-0.001 (0.99)	-0.007 (0.87)	0.013 (0.81)	0.075 (0.26)	0.047 (0.57)	0.071 (0.34)
	[0.05, 0.37]	[-0.29, 0.29]	[-0.09, 0.08]	[-0.10, 0.12]	[-0.06, 0.21]	[-0.12, 0.21]	[-0.08, 0.22]
> 1000	0.328 (0.005)	0.156 (0.70)	0.057 (0.43)	-0.037 (0.74)	0.129 (0.027)	-0.084 (0.54)	-0.190 (0.42)
	[0.10, 0.56]	[-0.64, 0.96]	[-0.08, 0.20]	[-0.26, 0.18]	[0.01, 0.24]	[-0.36, 0.19]	[-0.65, 0.27]
Household size	0.009 (0.15)	0.007 (0.77)	-0.002 (0.68)	-0.021 (0.11)	0.001 (0.85)	0.024 (0.009)	-0.003 (0.81)
	[-0.00, 0.02]	[-0.04, 0.05]	[-0.01, 0.01]	[-0.05, 0.01]	[-0.01, 0.02]	[0.01, 0.04]	[-0.03, 0.02]
Constant	0.692 (<0.0001)	0.803 (<0.0001)	0.605 (0.003)	0.718 (<0.0001)	0.950 (<0.0001)	0.950 (<0.0001)	1.150 (<0.0001)
	[0.39, 1.00]	[0.46, 1.15]	[0.21, 1.00]	[0.55, 0.89]	[0.79, 1.11]	[0.79, 1.11]	[0.83, 1.47]
Observations	1,049	182	4,098	1,607	1,034	1,034	331
R-squared	0.454	0.437	0.060	0.213	0.079	0.079	0.210
F-stat	0.183	13.67	3.754	21.89	2.835	2.835	2.028
Prob > F	0.67	<0.0001	<0.0001	<0.0001	0.0001	0.0001	0.008
F-stat d1=d2 d1=d3	0.150	0.165	0.333	1.292	0.550	0.550	0.0225
p>F d1=d2 d1=d3	0.86	0.85	0.72	0.28	0.58	0.58	0.98
F-stat d2=d3	0.183	0.0001	0.593	2.203	0.606	0.606	0.007
p>F d2=d3	0.67	0.99	0.44	0.14	0.44	0.44	0.94

Robust p values in parentheses

95% CI in brackets

This table presents the probabilities of receiving each health service associated with time since arriving to Brazil: less than 6 months; between 7 and 18 months; and, between 19 and 36.

All models are adjusted for sociodemographic characteristics of women.

F-stat: Tests whether there is no predictive relationship between the regressors and the outcome in our model. We reject the null hypothesis of no predictive relationship with values of Prob > F under 0.05.

p>F d1=d2 d1=d3 tests whether being in Brazil for 6 months or less (d1) is statistically different than being for more than 6 months (d2 & d3) for each outcome.

p>F d2=d3 tests whether being in Brazil for 7-18 months (d2) is statistically different than being for 19-36 months for each outcome.

Supplementary Table 8. Financial protection results: full POF sample of women from Roraima and Amazonas states

	FINANCIAL PROTECTION					
	Catastrophic health expenditures 10%		Catastrophic health expenditures 25%		Health Expenditures	
	(Model 1)	(Model 2)	(Model 3)	(Model 4)	(Model 5)	(Model 6)
Migrant	0.087 (<0.0001)	0.017 (0.36)	0.064 (<0.0001)	0.051 (<0.0001)	-55.794 (<0.0001)	-70.868 (<0.0001)
	[0.06, 0.12]	[-0.02, 0.05]	[0.05, 0.08]	[0.04, 0.07]	[-74.49, -37.10]	[-94.20, -47.54]
Migrant*Manaus		0.241 (<0.0001)		0.042 (0.004)		52.034 (0.0002)
		[0.19, 0.29]		[0.01, 0.07]		[24.66, 79.40]
Manaus	0.091 (<0.0001)	-0.043 (0.040)	0.021 (0.006)	-0.002 (0.70)	31.143 (0.003)	2.204 (0.89)
	[0.06, 0.12]	[-0.08, -0.00]	[0.01, 0.04]	[-0.01, 0.01]	[10.68, 51.60]	[-30.39, 34.80]
Observations	4,483	4,483	4,483	4,483	4,483	4,483
R-squared	0.053	0.084	0.036	0.038	0.089	0.096
F-stat	9.280	13.81	9.369	8.745	12.46	19.57
Prob > F	<0.0001	<0.0001	<0.0001	<0.0001	<0.0001	<0.0001

Robust p values in parentheses; 95% CI in brackets

Migrant*Manaus is the interaction term of migrant and Manaus. Refers to migrants living in the city of Manaus

Models 1 to 4 present the probabilities of incurring catastrophic health expenditures (10% or 25%) associated with being a migrant (vs local), being a migrant in Manaus (vs migrant in Boa Vista) or living in Manaus (vs living in Boa Vista). Models 5-6 show the difference in healthcare expenditures in R$ associated with being a migrant in Manaus, and living in Manaus (migrant and non-migrant). All models are adjusted for sociodemographic characteristics of women.

F-stat: Tests whether there is no predictive relationship between the regressors and the outcome in the models. We reject the null hypothesis of no predictive relationship with values of Prob > F smaller than 0.05.

Supplementary Table 9. Healthcare access, quality and health status results (general health): full PNS sample of women from Roraima and Amazonas states

	HEALTHCARE ACCESS, QUALITY, AND HEALTH STATUS					
	General health					
	Received care when sought		Did not pay for medications		Self-assessed good health	
	(Model 1)	(Model 2)	(Model 3)	(Model 4)	(Model 5)	(Model 6)
Migrant	0.225 (0.004)	0.220 (0.020)	0.089 (0.39)	0.095 (0.49)	-0.059 (0.07)	-0.015 (0.71)
	[0.07, 0.38]	[0.03, 0.40]	[-0.12, 0.29]	[-0.18, 0.37]	[-0.12, 0.00]	[-0.09, 0.06]
Migrant*Manaus		0.022 (0.88)		-0.028 (0.89)		-0.177 (0.013)
		[-0.28, 0.32]		[-0.43, 0.37]		[-0.32, -0.04]
Manaus	0.017 (0.74)	0.002 (0.99)	-0.425 (<0.0001)	-0.403 (0.020)	-0.057 (0.07)	0.050 (0.44)
	[-0.09, 0.12]	[-0.27, 0.28]	[-0.59, -0.26]	[-0.74, -0.06]	[-0.12, 0.00]	[-0.08, 0.18]
Observations	1,363	1,363	238	238	5,943	5,943
R-squared	0.230	0.230	0.314	0.314	0.049	0.056
F-stat	2.021	2.495	10.63	12.43	4.748	5.963
Prob > F	0.009	0.0007	<0.0001	<0.0001	<0.0001	<0.0001

Robust p values in parentheses; 95% CI in brackets

Migrant*Manaus is the interaction term of migrant and Manaus. Refers to migrants living in the city of Manaus

This table presents the probabilities of receiving each health service associated with being a migrant (vs local), being a migrant in Manaus (vs migrant in Boa Vista) or living in Manaus (vs living in Boa Vista).

All models are adjusted for sociodemographic characteristics of women.

F-stat: Tests whether there is no predictive relationship between the regressors and the outcome in the models. We reject the null hypothesis of no predictive relationship with values of Prob > F smaller than 0.05.

Supplementary Table 10. Healthcare access and quality results (sexual and reproductive health): full PNS sample of women from Roraima and Amazonas states

	HEALTHCARE ACCESS, QUALITY AND HEALTH STATUS							
	Sexual and reproductive health							
	Pap smear in last 3 years		Antenatal care consultation		Timely pap smear results		Antenatal care as per guidelines	
	(Model 5)	(Model 6)	(Model 7)	(Model 8)	(Model 9)	(Model 10)	(Model 11)	(Model 12)
Migrant	-0.410 (<0.0001)	-0.470 (<0.0001)	-0.001 (0.98)	0.009 (0.91)	0.126 (0.033)	0.196 (0.011)	0.090 (0.23)	0.146 (0.15)
	[-0.47, -0.35]	[-0.55, -0.39]	[-0.13, 0.13]	[-0.16, 0.18]	[0.01, 0.24]	[0.04, 0.35]	[-0.06, 0.24]	[-0.06, 0.35]
Migrant*Manaus		0.203 (0.003)		-0.039 (0.66)		-0.186 (0.13)		-0.203 (0.09)
		[0.07, 0.34]		[-0.21, 0.13]		[-0.43, 0.05]		[-0.44, 0.03]
Manaus	0.125 (0.0005)	0.001 (0.99)	0.059 (0.07)	0.084 (0.223)	0.043 (0.47)	0.133 (0.22)	0.118 (0.031)	0.245 (0.022)
	[0.05, 0.19]	[-0.11, 0.11]	[-0.00, 0.12]	[-0.05, 0.22]	[-0.07, 0.16]	[-0.08, 0.35]	[0.01, 0.23]	[0.03, 0.46]
Observations	2,148	2,148	468	468	1,471	1,471	425	425
R-squared	0.208	0.216	0.096	0.097	0.134	0.142	0.089	0.102
F-stat	24.90	25.08	1.803	1.935	6.974	5.940	3.103	3.027
Prob > F	<0.0001	<0.0001	0.028	0.014	<0.0001	<0.0001	<0.0001	<0.0001

Robust p values in parentheses

95% CI in brackets

Migrant*Manaus is the interaction term of migrant and Manaus. Refers to migrants living in the city of Manaus

This table presents the probabilities of receiving each health service associated with being a migrant (vs local), being a migrant in Manaus (vs migrant in Boa Vista) or living in Manaus (vs living in Boa Vista).

All models are adjusted for sociodemographic characteristics of women.

F-stat: Tests whether there is no predictive relationship between the regressors and the outcome in the models. We reject the null hypothesis of no predictive relationship with values of Prob > F smaller than 0.05.

Supplementary Table 11. Financial protection results: marginal effects from logit estimations

	FINANCIAL PROTECTION			
	Catastrophic health expenditure 10%		Catastrophic health expenditure 25%	
	(Model 1)	(Model 2)	(Model 3)	(Model 4)
Migrant	0.096 (<0.0001)	0.007 (0.81)	0.066 (<0.0001)	0.055 (0.0008)
	[0.06, 0.13]	[-0.05, 0.07]	[0.05, 0.08]	[0.02, 0.09]
Migrant*Manaus		0.204 (0.0001)		0.028 (0.56)
		[0.10, 0.31]		[-0.06, 0.12]
Manaus	0.066 (0.0005)	-0.073 (0.06)	0.021 (0.005)	-0.006 (0.90)
	[0.03, 0.10]	[-0.15, 0.00]	[0.01, 0.04]	[-0.09, 0.08]
Observations	3,439	3,439	3,439	3,439

Robust p values in parentheses

95% CI in brackets

Migrant*Manaus is the interaction term of migrant and Manaus. Refers to migrants living in the city of Manaus

Models 1 to 4 present logistic regression marginal effects of incurring catastrophic health expenditures (10% or 25%) associated with being a migrant (vs local), being a migrant in Manaus (vs migrant in Boa Vista) or living in Manaus (vs living in Boa Vista). All models are adjusted for sociodemographic characteristics of women.

F-stat: Tests whether there is no predictive relationship between the regressors and the outcome in the models. We reject the null hypothesis of no predictive relationship with values of Prob > F smaller than 0.05.

Supplementary Table 12. Healthcare access, quality and health status results (general health): marginal effects from logit estimations

	ACCESS TO CARE, QUALITY AND HEALTH STATUS					
	General health					
	Received care when sought		Did not pay for medications		Good self-assessed health	
	(Model 1)	(Model 2)	(Model 3)	(Model 4)	(Model 5)	(Model 6)
Migrant	0.200 (<0.0001)	0.179 (<0.0001)	0.115 (0.23)	0.025 (0.85)	-0.112 (0.002)	-0.072 (0.23)
	[0.14, 0.26]	[0.11, 0.25]	[-0.07, 0.30]	[-0.24, 0.29]	[-0.18, -0.04]	[-0.19, 0.05]
Migrant*Manaus		0.087 (0.27)		0.230 (0.20)		-0.100 (0.31)
		[-0.07, 0.24]		[-0.12, 0.58]		[-0.29, 0.09]
Manaus	-0.049 (0.25)	-0.073 (0.19)	-0.371 (<0.0001)	-0.539 (0.0002)	-0.073 (0.06)	-0.020 (0.81)
	[-0.13, 0.03]	[-0.18, 0.04]	[-0.45, -0.29]	[-0.82, -0.26]	[-0.15, 0.00]	[-0.18, 0.14]
Observations	1,041	1,041	182	182	4,113	4,113

Robust p values in parentheses

95% CI in brackets

Migrant*Manaus is the interaction term of migrant and Manaus. Refers to migrants living in the city of Manaus

This table presents logistic regression marginal effects of receiving each health service associated with being a migrant (vs local), being a migrant in Manaus (vs migrant in Boa Vista) or living in Manaus (vs living in Boa Vista).

All models are adjusted for sociodemographic characteristics of women.

F-stat: Tests whether there is no predictive relationship between the regressors and the outcome in the models. We reject the null hypothesis of no predictive relationship with values of Prob > F smaller than 0.05.

Supplementary Table 13. Healthcare access and quality results (sexual and reproductive health): marginal effects from logit estimations

	HEALTHCARE ACCESS, QUALITY AND HEALTH STATUS							
	Sexual and reproductive health							
	Pap smear in last 3 years		Antenatal care consultation		Timely pap smear results		Antenatal care as per guidelines	
	(Model 7)	(Model 8)	(Model 9)	(Model 10)	(Model 11)	(Model 12)	(Model 13)	(Model 14)
Migrant	-0.349 (<0.0001)	-0.394 (<0.0001)	0.128 (0.05)	0.180 (0.0008)	0.076 (0.33)	0.060 (0.50)	0.139 (0.07)	0.211 (0.0007)
	[-0.43, -0.27]	[-0.51, -0.28]	[-0.00, 0.26]	[0.08, 0.29]	[-0.08, 0.23]	[-0.12, 0.24]	[-0.01, 0.29]	[0.09, 0.33]
Migrant*Manaus		0.117 (0.37)		-0.480 (0.0002)		0.031 (0.77)		-0.466 (0.0001)
		[-0.14, 0.38]		[-0.73, -0.23]		[-0.18, 0.24]		[-0.69, -0.24]
Manaus	0.108 (0.037)	0.031 (0.80)	0.175 (0.008)	0.483 (<0.0001)	-0.016 (0.82)	-0.027 (0.78)	0.211 (0.019)	0.450 (<0.0001)
	[0.01, 0.21]	[-0.21, 0.27]	[0.05, 0.30]	[0.26, 0.70]	[-0.15, 0.12]	[-0.22, 0.16]	[0.03, 0.39]	[0.25, 0.65]
Observations	1,615	1,615	350	350	1,039	1,039	328	328

Robust p values in parentheses

95% CI in brackets

Migrant*Manaus is the interaction term of migrant and Manaus. Refers to migrants living in the city of Manaus

This table presents logistic regression marginal effects of receiving each health service associated with being a migrant (vs local), being a migrant in Manaus (vs migrant in Boa Vista) or living in Manaus (vs living in Boa Vista).

All models are adjusted for sociodemographic characteristics of women.

F-stat: Tests whether there is no predictive relationship between the regressors and the outcome in the models. We reject the null hypothesis of no predictive relationship with values of Prob > F smaller than 0.05.

Supplementary Table 14. Financial protection results: estimations accounting for survey weights

	FINANCIAL PROTECTION					
	**Catastrophic health expenditure 10%**		**Catastrophic health expenditure 25%**		**Health Expenditures (R$)**	
	**(Model 1)**	**(Model 2)**	**(Model 3)**	**(Model 4)**	**(Model 5)**	**(Model 6)**
Migrant	0.090 (<0.0001)	0.011 (0.66)	0.066 (<0.0001)	0.054 (<0.0001)	-63.093 (<0.0001)	-84.062 (<0.0001)
	[0.05, 0.13]	[-0.04, 0.06]	[0.05, 0.08]	[0.03, 0.07]	[-82.19, -44.00]	[-111.36, -56.77]
Migrant*Manaus		0.249 (<0.0001)	0.222 (<0.0001)		0.034 (0.0449)	
		[0.16, 0.29]		[0.00, 0.07]		[27.25, 90.62]
Manaus	0.060 (0.001)	-0.050 (0.05)	0.016 (0.06)	-0.001 (0.83)	21.105 (0.031)	-8.265 (0.62)
	[0.02, 0.10]	[-0.10, 0.00]	[-0.00, 0.03]	[-0.01, 0.01]	[1.88, 40.33]	[-41.20, 24.67]
	3,439	3,439	3,439	3,439	3,439	3,439
R-squared	0.042	0.073	0.038	0.040	0.133	0.144
F-stat	6.565	10.08	7.057	6.548	11	15.58
Prob > F	<0.0001	<0.0001	<0.0001	<0.0001	<0.0001	<0.0001

Robust p values in parentheses

95% CI in brackets

Models 1 to 4 present the probabilities of incurring catastrophic health expenditures (10% or 25%) associated with being a migrant (vs local), being a migrant in Manaus (vs migrant in Boa Vista) or living in Manaus (vs living in Boa Vista). Models 5-6 show the difference in healthcare expenditures in R$ associated with being a migrant, being a migrant in Manaus, and living in Manaus (migrant and non-migrant). All models are adjusted for sociodemographic characteristics of women.
F-stat: Tests whether there is no predictive relationship between the regressors and the outcome in the models. We reject the null hypothesis of no predictive relationship with values of Prob > F smaller than 0.05.

Supplementary Table 15. Healthcare access, quality and health status results (general health): estimations accounting for survey weights

	ACCESS TO CARE, QUALITY OF CARE AND HEALTH STATUS					
	**General health**					
	**Sought and received care**		**Publicly provided meds**		**Good self-assessed health**	
	**(Model 1)**	**(Model 2)**	**(Model 3)**	**(Model 4)**	**(Model 5)**	**(Model 6)**
Migrant	0.241 (0.004)	0.256 (0.033)	0.143 (0.18)	0.093 (0.49)	-0.099 (0.016)	-0.059 (0.33)
	[0.08, 0.41]	[0.02, 0.49]	[-0.07, 0.35]	[-0.17, 0.36]	[-0.18, -0.02]	[-0.18, 0.06]
Migrant*Manaus		-0.040 (0.82)	-0.041 (0.81)		0.114 (0.52)	
		[-0.38, 0.29]		[-0.24, 0.46]		[-0.28, 0.07]
Manaus	-0.064 (0.42)	-0.043 (0.78)	-0.548 (<0.0001)	-0.624 (<0.0001)	-0.080 (0.06)	-0.030 (0.69)
	[-0.22, 0.09]	[-0.34, 0.26]	[-0.74, -0.36]	[-0.89, -0.36]	[-0.16, 0.00]	[-0.18, 0.12]
Observations	1,053	1,053	184	184	4,113	4,113
R-squared	0.403	0.406	0.507	0.508	0.067	0.071
F-stat	1.130	1.611	28.03	64.39	4.136	5.146
Prob > F	0.32	0.05	<0.0001	<0.0001	<0.0001	<0.0001

Robust p values in parentheses

95% CI in brackets

Migrant*Manaus is the interaction term of migrant and Manaus. Refers, migrants living in the city of Manaus
This table presents the probabilities of receiving each health service associated with being a migrant (vs local), being a migrant in Manaus (vs migrant in Boa Vista) or living in Manaus (vs living in Boa Vista).
All models are adjusted for sociodemographic characteristics of women.
F-stat: Tests whether there is no predictive relationship between the regressors and the outcome in the models. We reject the null hypothesis of no predictive relationship with values of Prob > F smaller than 0.05.

Supplementary Table 16. Healthcare access and quality results (sexual and reproductive health): estimations accounting for survey weights

	HEALTHCARE ACCESS, QUALITY, AND HEALTH STATUS							
	Sexual and reproductive health							
	Pap smear in last 3 years		Antenatal care consultation		Timely pap smear results		Antenatal care as per guidelines	
	(Model 7)	(Model 8)	(Model 9)	(Model 10)	(Model 11)	(Model 12)	(Model 13)	(Model 14)
Migrant	-0.370 (<0.0001)	-0.450 (<0.0001)	0.098 (0.43)	0.284 (0.14)	0.120 (0.19)	0.080 (0.45)	0.163 (0.16)	0.377 (0.039)
	[-0.45, -0.29]	[-0.56, -0.34]	[-0.14, 0.34]	[-0.09, 0.66]	[-0.06, 0.30]	[-0.13, 0.29]	[-0.07, 0.39]	[0.02, 0.74]
Migrant*Manaus		0.194 (0.042)		-0.442 (0.05)		0.083 (0.56)		-0.502 (0.020)
		[0.01, 0.38]		[-0.89, 0.01]		[-0.19, 0.36]		[-0.93, -0.08]
Manaus	0.112 (0.033)	0.010 (0.91)	0.182 (0.007)	0.436 (0.014)	-0.046 (0.59)	-0.083 (0.54)	0.172 (0.028)	0.446 (0.008)
	[0.01, 0.21]	[-0.16, 0.18]	[0.05, 0.31]	[0.09, 0.78]	[-0.21, 0.12]	[-0.35, 0.18]	[0.02, 0.33]	[0.12, 0.78]
Observations	1,615	1,615	374	374	1,039	1,039	331	331
R-squared	0.236	0.244	0.235	0.289	0.161	0.163	0.260	0.327
F-stat	22.44	25.65	1.612	1.887	1.372	1.504	1.972	2.079
Prob > F	<0.0001	<0.0001	0.06	0.018	0.15	0.09	0.015	0.008

Robust p values in parentheses

95% CI in brackets

Migrant*Manaus is the interaction term of migrant and Manaus. Refers, migrants living in the city of Manaus
This table presents the probabilities of receiving each health service associated with being a migrant (vs local), being a migrant in Manaus (vs migrant in Boa Vista) or living in Manaus (vs living in Boa Vista).
All models are adjusted for sociodemographic characteristics of women.
F-stat: Tests whether there is no predictive relationship between the regressors and the outcome in the models. We reject the null hypothesis of no predictive relationship with values of Prob > F smaller than 0.05.

References

1. Heckathorn DD. Respondent-driven sampling: a new approach, the study of hidden populations. Social problems. 1997 May 1;44(2):174—99.
2. Szwarcwald CL, de Souza Júnior PRB, Damacena GN, Junior AB, Kendall C. Analysis of Data Collected by RDS Among Sex Workers in 10 Brazilian Cities, 2009: Estimation of the Prevalence of HIV, Variance, and Design Effect. JAIDS J Acquir Immune Defic Syndr. 15 de agosto de 2011;57:S129.
3. Handcock MS, Fellows IF, Gile KJ. RDS Analyst: Software for the Analysis of Respondent-Driven Sampling Data, Version 0.42 [Internet]. 2014 [citado 4 de julho de 2022]. Disponível em: http://hpmrg.org
4. Leal MC, Gomes TD, Santos Y, Queiroz RS, Fonseca PA, Szwarcwald C and Riggirozzi P. Migration process of Venezuelan women, Brazil: Living conditions and use of health services in Manaus and Boa Vista, 2018–2021. (Forthcoming BMC Public Health).
5. Aloe AM, Becker BJ. An effect size for regression predictors in meta-analysis. Journal of Educational and Behavioral Statistics. 2012 Apr;37(2):278-97.
